# Equivalent carbon number-based targeted odd-chain fatty acyl lipidomics reveals triacylglycerol profiling in clinical colon cancer

**DOI:** 10.1016/j.jlr.2023.100393

**Published:** 2023-05-29

**Authors:** Jiangang Zhang, Shuai Yang, Jingchun Wang, Yanquan Xu, Huakan Zhao, Juan Lei, Yu Zhou, Yu Chen, Lei Wu, Yongsheng Li

**Affiliations:** 1 Department of Medical Oncology, Chongqing University Cancer Hospital, Chongqing, China; 2 Department of Pathology, The 958th Hospital, Southwest Hospital, Army Medical University, Chongqing, China; 3 Department of Gastroenterology, Xinqiao Hospital, Army Medical University, Chongqing China; 4 Clinical Medicine Research Center, Xinqiao Hospital, Army Medical University, Chongqing China

**Keywords:** colon cancer, equivalent carbon number, LC-MS, lipidomics, odd-chain FAs

## Abstract

Odd-chain FAs (OCFAs) are present in very low level at nearly 1% of total FAs in human plasma, and thus, their functions were usually ignored. Recent epidemiological studies have shown that OCFAs are inversely associated with a variety of disease risks. However, the contribution of OCFAs incorporated into complex lipids remains elusive. Here, we developed a targeted odd-chain fatty acyl-containing lipidomics method based on equivalent carbon number and retention time prediction. The method displayed good reproducibility and robustness as shown by peak width at half height within 0.7 min and coefficient of variation under 20%. A total number of 776 lipid species with odd-chain fatty acyl residues could be detected in the ESI mode of reverse-phase LC-MS, of which 309 lipids were further validated using multiple reaction monitoring transitions. Using this method, we quantified odd-chain fatty acyl-containing lipidome in tissues from 12 colon cancer patients, revealing the remodeling of triacylglycerol. The dynamics of odd-chain fatty acyl lipids were further consolidated by the association with genomic and proteomic features of altered catabolism of branched-chain amino acids and triacylglycerol endogenous synthesis in colon cancer. This lipidomics approach will be applicable for screening of dysregulated odd-chain fatty acyl lipids, which enriches and improves the methods for diagnosis and prognosis evaluation of cancer using lipidomics.

## Graphical Abstract

**Figure:**
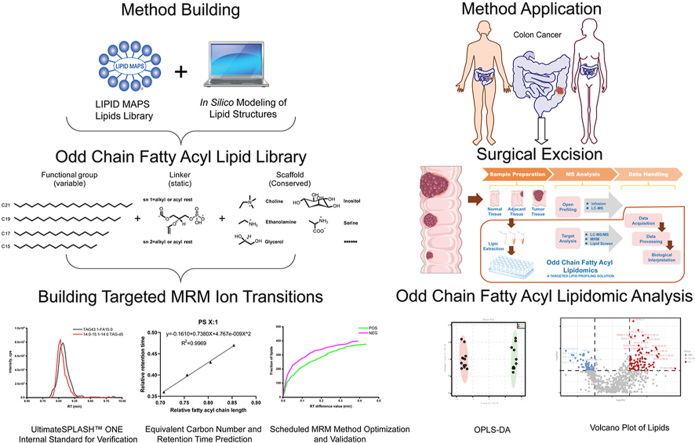

FAs are categorized as even- and odd-chain subclass based on its aliphatic chain length. They exist extensively in the body of beings from the low-life form such as bacteria and fungi (1, 2) to the structure and body fluid of animals and plants (3, 4). The paradigm of research into FA metabolism was conducted primarily on even-chain FAs (C2-C26) in humans (5). By contrast, the odd-chain FAs (OCFAs, mainly C15:0, C17:1, and C17:0) are present in very low abundance at nearly 1% as a percentage of total C12-C20 FAs in human plasma (6). Whereas, the revolutionary development of LC-MS enabled the detection of trace amount of OCFA in human bloods and tissues, successively added C19:1 (7) and C23:0 (8) to the lipids.

OCFAs are normally recognized as exogenous source originated from dietary ruminant fat since ruminants can synthesize OCFAs through bacterial fermentation in the rumen (9). OCFAs absorbed from the intestine can be subsequently utilized in the mammary gland, resulting in its incorporation into milk fat (10). Meanwhile, accumulating evidences suggest that OCFAs can be transformed endogenously from peroxisomal α-oxidation of long-chain FAs (9, 11) or from de novo lipogenesis in the liver and adipose tissue using propionate-derived propionyl-CoA, instead of acetyl-CoA or gut microbiota-derived short-chain FAs, as primer (12). In addition, other nutritional component catabolism such as methionine (13), threonine (14), branched-chain amino acids (BCAAs) (14) and cholesterol (15) can also produce propionyl-CoA for OCFA synthesis. However, controversy referring to the contributions from nonruminant gut microbiota or from endogenous biosynthesis of OCFAs remains existed.

As the basic building blocks of complex lipids, FAs are diversely incorporated into a variety of lipids, exerting significant roles in lipid metabolism (3). As a result, altered composition in different lipid species, also known as lipidome (16, 17), is often viewed as preliminary proof for physiological and pathological perturbations. Accordingly, lipidomics is a powerful technique to profiling large amount of lipid fluctuation in health and diseases (18, 19), which massively assisted the researchers to uncover the mysterious lipids.

Metabolic reprogramming is the hallmark of cancer, and aberrant lipid metabolism contributes to the carcinogenesis and metastasis of colon cancer (20). Recent epidemiological studies have shown that OCFAs are inversely associated with colorectal cancer (CRC) risk (21, 22). A cross-sectional analysis in the EPIC-InterAct study confirmed that higher abundances of total C15:0 and C17:0 are the leading signatures of lower metabolic risk (23, 24). Nonetheless, these observations from prospective or epidemiological studies only demonstrated the association other than causative relationship between OCFAs and CRC. Moreover, the dynamics and function of odd-chain fatty acyl lipidome during the progression of colon cancer remain scarcely explored, which poses a hurdle to the biomarker screening and functional lipid deciphering.

Herein, to gain insight into the potentially dynamic variation of odd-chain fatty acyl lipid composition in colon cancer, we developed a quantitative and reverse LC-MS-based scheduled multiple reaction monitoring (sMRM) lipidomics approach coupled with equivalent carbon number (ECN) and retention time (RT) prediction model to robustly analyze a wide range of odd-chain fatty acyl lipid species in malignant and matched nonmalignant tissues. We also aimed to reveal alteration of triglyceride composition during the progression of colon cancer. Further linkage corroborated that the genomic and proteomic feature of dysfunctional lipid metabolism was analyzed. These results provide the first comprehensive signature of the odd-chain fatty acyl lipidomic landscape and enhance our understanding of these lipids in colon cancer.

## Materials and Methods

### Chemicals and standards

Acetonitrile, dichloroform (DCM), isopropanol, and methanol (MEOH) were purchased from Honeywell (NJ). All solvents were HPLC grade. Deionized water was provided by a Millipore Milli-Q purification system (Bedford, MA). Ammonium acetate was purchased from Sigma-Aldrich (Taufkirchen, Germany). The authentic standards of triacylglycerol (TAG) including tritridecanoin (C13:0, CAS#26536-12-9), tripentadecanoin (C15:0, CAS#7370-46-9), triheptadecanoin (C17:0, CAS#2438-40-6), and tritricosanoin (C23:0, CAS#86850-72-8) were bought from Shanghai ANPEL Experimental Technology Co, Ltd (https://www.labsci.com.cn/). UltimateSPLASH™ ONE (catalog no.: 330820L) for lipidomic analysis is a commercialized mixture of deuterium-labeled internal standard (IS) provided by Avanti Polar Lipids, Inc (AL). The mixture consists of the 69 deuterium-labeled ISs in 1:1 dichloromethane:MEOH solution (1.2 ml per vial). Detailed ISs are listed in supplemental Table S1. The isotope-labeled standards were provided at defined concentrations by Avanti Polar Lipids and used without further purification or validation. Certificate of analysis for UltimateSPLASH™ ONE is available at https://avantilipids.com/.

### Human tissue samples

Colon tumor tissues with matching normal samples including distant normal tissues and adjacent tissues were obtained from previously untreated patients who had undergone colectomy (Chongqing University Cancer Hospital, China) in Chongqing Key Laboratory of Translational Research for Cancer Metastasis and Individualized Treatment (Chongqing University Cancer Hospital, China). Clinical profile of patients are provided in supplemental Tables S2 and S3. Samples were snap frozen and stored at −80°C for lipid and protein extractions. Normal and tumor tissues were identified by histologic analysis of adjacent tissue, Gleason scores were determined, and the percentage of cancer was estimated. All tumor samples used for lipidomics were verified to contain at least 75% colon adenocarcinoma (COAD) by histologic examination. The study was also performed according to the Declaration of Helsinki principles, and the utilization of clinical specimens was approved by the local Ethical Committee of Chongqing University Cancer Hospital.

### Mouse tissue samples

All mouse experiments were performed with the approval of the local Ethical Committee of Chongqing University Cancer Hospital. C57BL/6 background mice were maintained on standard chow diet, and water was available ad libitum in a vivarium with a 12 h light-dark cycle at 22°C. Mice were sacrificed after anesthetizing, and tissues were collected for further analysis.

### Sample preparation

In brief, before weighing, the tissue fluid or blood was dried with filter paper. A 50.0 mg of tissue was then weighed and placed into a 2 ml round bottom centrifuge tube, followed by adding 500 μl normal saline (0.85% normal saline preparation: 0.85 g NaCl was added to a 100 ml deionized water, and then 40 μl glacial acetic acid was added). Grinding was next performed by adding two large magnetic beads (small magnetic beads were only used to homogenize liver, kidney, brain, and mucous membrane tissues, and generally two small magnetic beads were put) into the sample tube and transferring it into the homogenizer for homogenizing twice, each lasts for 30 s and the interval is 10 s. For the muscles or connective tissues, one or two more times were needed to fulfill sufficient homogenized. The homogenates were subsequently centrifuged at 12,000 rpm for 15 min at 4°C and used for further lipid extraction.

### Lipid extraction

The lipid extraction protocol represents a modified Bligh and Dyer procedure (25). Briefly, the supernatant of tissue homogenate (50 μl) and 5 μl of the IS mixture were added into the glass tube, followed by adding 950 μl H_2_O, 2 ml MEOH, and 0.9 ml DCM. Gently, the tube was vibrated for 5 s, and no delamination occurs at this time. If it does, 50 μl more MEOH was added. The tube was next placed at room temperature for 30 min and then by adding 1 ml H_2_O and 0.9 ml DCM successively. The tube was slight vibrated for 5 s and then centrifuged at 2000 rpm for 15 min until the liquid delaminates (please note: excessive vibration can lead to poor delamination). The lower liquid phase was pipetted to a new glass tube, and 1.8 ml DCM was added to the original tube for the second extraction. Followed the above steps, the second lower liquid phase extracts were combined with the first one. Then, the extract was dried with stable nitrogen stream. A 100 μl working solution was added into the tube to dissolve it (working solution composition: DCM containing 10 mM ammonium acetate:MEOH, 1:1, v/v). The sample was next shook on a bench vibrator to mix well and transferred to a 200 μl LC-MS insert vial. The vial was placed into a 1.5 ml plastic Eppendorf tube and centrifuged at 16,000 rpm for 10 min. A 10 μl of each sample was took into a tube of quality control (QC) for subsequent use as a control and mixed well. Finally, the lipid extracts in vials were kept in the −80°C freezer until LC-MS/MS analysis. On the day of analysis, LC–MS vials were taken out of the freezer, thawed at room temperature for 30 min, sonicated in ice-cold water for 15 min, and injected into LC-MS/MS machine.

### Reverse phase-UPLC-ESI-MS/MS-based lipidomics

The reverse phase-UPLC-MS/MS analysis was performed on the Acquity UPLC system (Waters Corporation, Milford, MA) connected to the AB/SCIEX QTRAP 6500 mass spectrometer and Exion UHPLC-AB/SCIEX QTRAP 6500+ (Applied Biosystems, Foster City, CA). The Analyst 1.6.3 software (AB SCIEX) was used for the data acquisition. The Acquity UPLC BEH C18 column (2.1 × 50 mm; 1.7 μm; Waters Corporation) was used for the LC separation. Mobile phases A (MEOH:acetonitrile:H_2_O 1:1:1, v/v/v, +5 mM ammonium acetate) and B (isopropanol, +5 mM ammonium acetate) were used for both positive and negative ionizations. The following gradient was applied: 0 min 20% B, 1 min 20% B, 2.5 min 40% B, 4 min 60% B, 14 min 90% B, 15 min 90% B, 15.1 min 20% B, and 17 min 20% B (total runtime of 17 min). The column temperature was maintained at 40°C. The flow rate was set to 0.2 ml/min for the Acquity UPLC system and 0.3 ml/min for Exion UHPLC, and the sample injection volume was 2 μl. All samples were kept at 4°C in sample trays throughout the analysis. In negative ESI mode, ion spray voltage, curtain gas, nebulizer gas (GS1), and turbo gas (GS2) were set at −4,500 V, 35 psi, 60 psi, and 60 psi, respectively. The turbo ion spray source temperature was 600°C. In positive ESI mode, ion spray voltage, curtain gas, GS1, and GS2 were set at 5,200 V, 40 psi, 55 psi, and 55 psi, respectively. The turbo ion spray source temperature was 350°C. The collision gas (collision-activated dissociation) was set as medium in both positive and negative modes.

Lipids were analyzed using sMRM. Mass spectrometer parameters, including the declustering potentials, entrance potential, collision energy, and collision cell exit potential, were optimized for each subclass of lipid species. Nitrogen was employed as the collision gas. The MRM list is provided in supplemental Tables S4 and S5. Quantitative data were extracted by using the MultiQuant software 3.0.2 (AB SCIEX). The data were manually curated to ensure that the software integrated the right peaks. Peak areas were normalized to the peak areas of IS. The data quality was assessed using the following criteria: MRM transitions kept for the analysis had to satisfy coefficient of variation (CV) measured across the QC injections <20%, blank samples consisting of only diluent were measured to determine background signals. Only in lipid species with an intensity >5-fold, the intensity of the blank was considered true signal.

### Method validation and QC

The reverse-phase-UPLC/MS method was validated as previously published (26). Solvent blanks and QC samples were regularly measured after each six samples. For the QC samples, the pooled extracted samples were used and aliquoted. In order to assess the instrumental state, the instrument stability, and sample preparation quality, the signal response of the IS in all samples was monitored during the whole sequence. The signal responses of selected lipids were plotted against the number of measured samples, which allowed the visualization of outliers because of sample preparation or instrumental failures. Furthermore, principal component analysis (PCA) for the lipidomic profiles in all samples was performed to review for outliers.

### Genomic and proteomic data availability

RNA-Seq data of The Cancer Genome Atlas were downloaded from UCSC Xena dataset (https://xenabrowser.net/datapages/) in the form of HTSeq-FPKM. The COAD data were then filtered, including 469 primary tumor samples and 41 matched normal samples. The proteomics data of COAD were downloaded from the Clinical Proteomic Tumor Analysis Consortium database (https://cptac-data-portal.georgetown.edu/) with the study ID of PDC000116 in the experiment types of TMT10, including 97 primary tumor samples and 100 normal samples. The relative expression matrix in transcriptomes and proteomes of the enzymes in odd-chain fatty acyl lipid metabolism was extracted and plotted in heatmap by using *ComplexHeatmap* (version 2.6.2) R package.

### Statistical analysis

The relative abundance files (supplemental Table S6) were uploaded into MetaboAnalyst (https://www.metaboanalyst.ca/) in the format of concentrations, data were filtered by interquantile range, and was normalized by log transformation and pareto scaling. Normalized data were used for further analysis including the data used in SIMCA software, version 14.1 (Umetrics, Umeå, Sweden). SIMCA was used to perform the unsupervised PCA with unclassified samples, and the supervised orthogonal partial least squares-discriminant analysis (OPLS-DA) with the known sample classification. Only scatter plots of the first and second components were presented in PCA score plots. OPLS-DA separated samples based on the known classes and was used for prediction. The model fit was determined by the evaluation of R2, which describes the variation of variables (lipid species) explained by the model. The insight into the prediction ability of the model was described by Q2 and was estimated using 7-fold crossvalidation in SIMCA software. PCA plot was evaluated for outliers, errors in measurements as for the separation of sample types. Afterward, OPLS-DA was performed to discriminate between CC-A versus CC-B and CC-B versus CC-C. To evaluate the statistical significance, a two-sided two-sample *t*-test assuming unequal variances (Welch test) was performed for healthy and cancer samples in MetaboAnalyst. *P* values <0.05 were considered to indicate the statistical significance. The Bonferroni approach was applied to all *P* values for the multiple testing correction. Furthermore, the parameter of variable importance of projection (VIP) was evaluated for each statistical OPLS-DA model using the SIMCA software. Finally, only lipid species with *P* values <0.05, VIP values >1, and fold changes (FCs) ≥20% for relative abundance were considered as statistically important and reported in supplemental Tables S6–S9. For verification of the data processing, statistical analysis, and results, data were crosschecked and independently reprocessed or evaluated by applying the online metabolomics platform MetaboAnalyst.

**Fig. 1:**
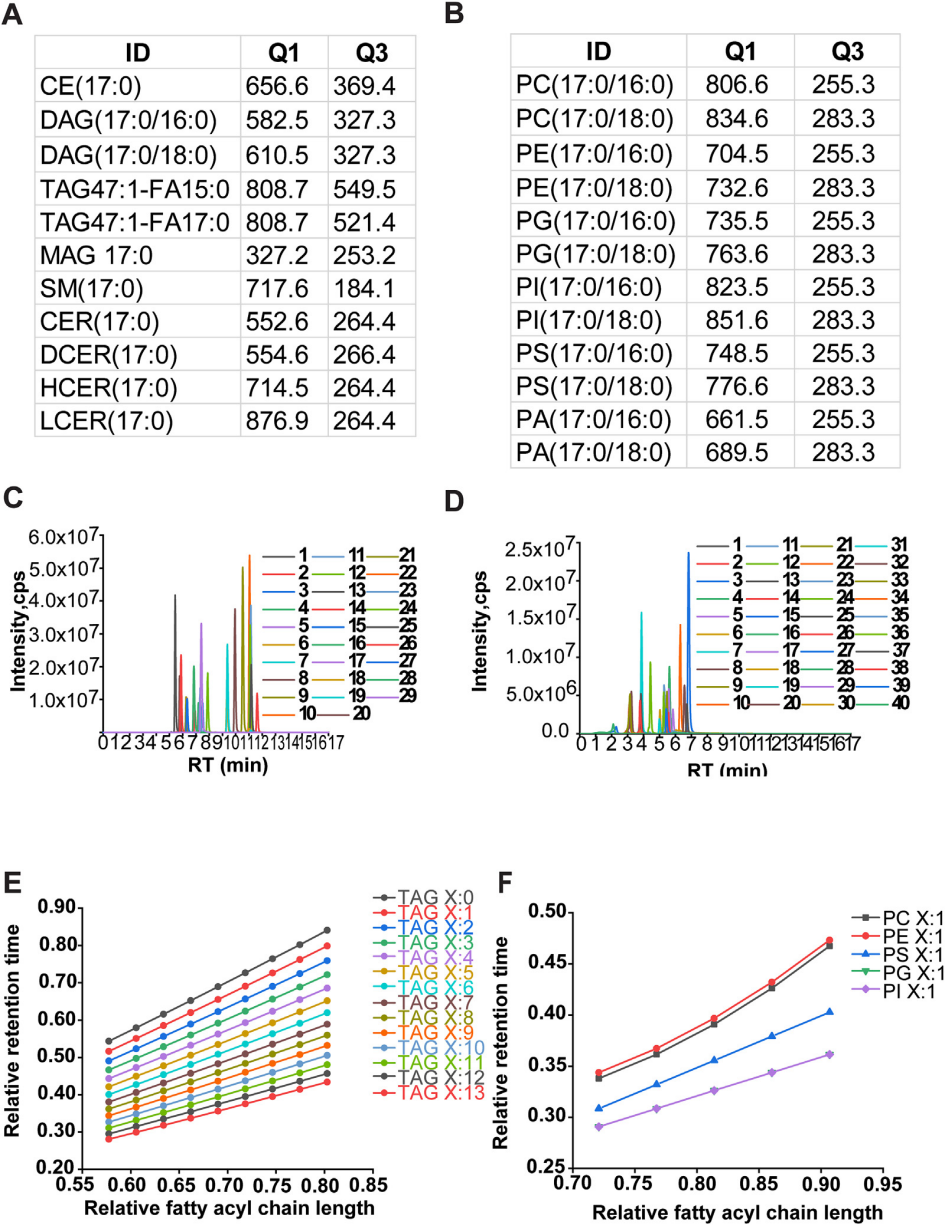
Targeted sMRM ion pairs of lipids based on their dissociation pattern in ESI and ECN intraclass RT prediction. MRM ion pairs in positive (A) and negative (B) ESI mode, Q1 *m/z* represents precursor ion, Q3 *m/z* represents product ion; deduced MRM of ISs detected in positive (C) and negative (D) ESI mode, lipid ID of their corresponding peak no. is provided in supplemental Table S1, x represents RT (min), y represents intensity (cps); ECN intraclass RT prediction of lipids in positive (E) and negative (F) ESI mode, binominal regression of each lipid subclass is provided in supplemental Table S10. X represents carbon chain length, binomial curve fitting was used via y = a + bx+ cx^2^, x represents relative carbon number (CN) (CN/CN_max_), y represents relative RT (RT/LC elution time), and R represents correlation coefficient.

**Fig. 2:**
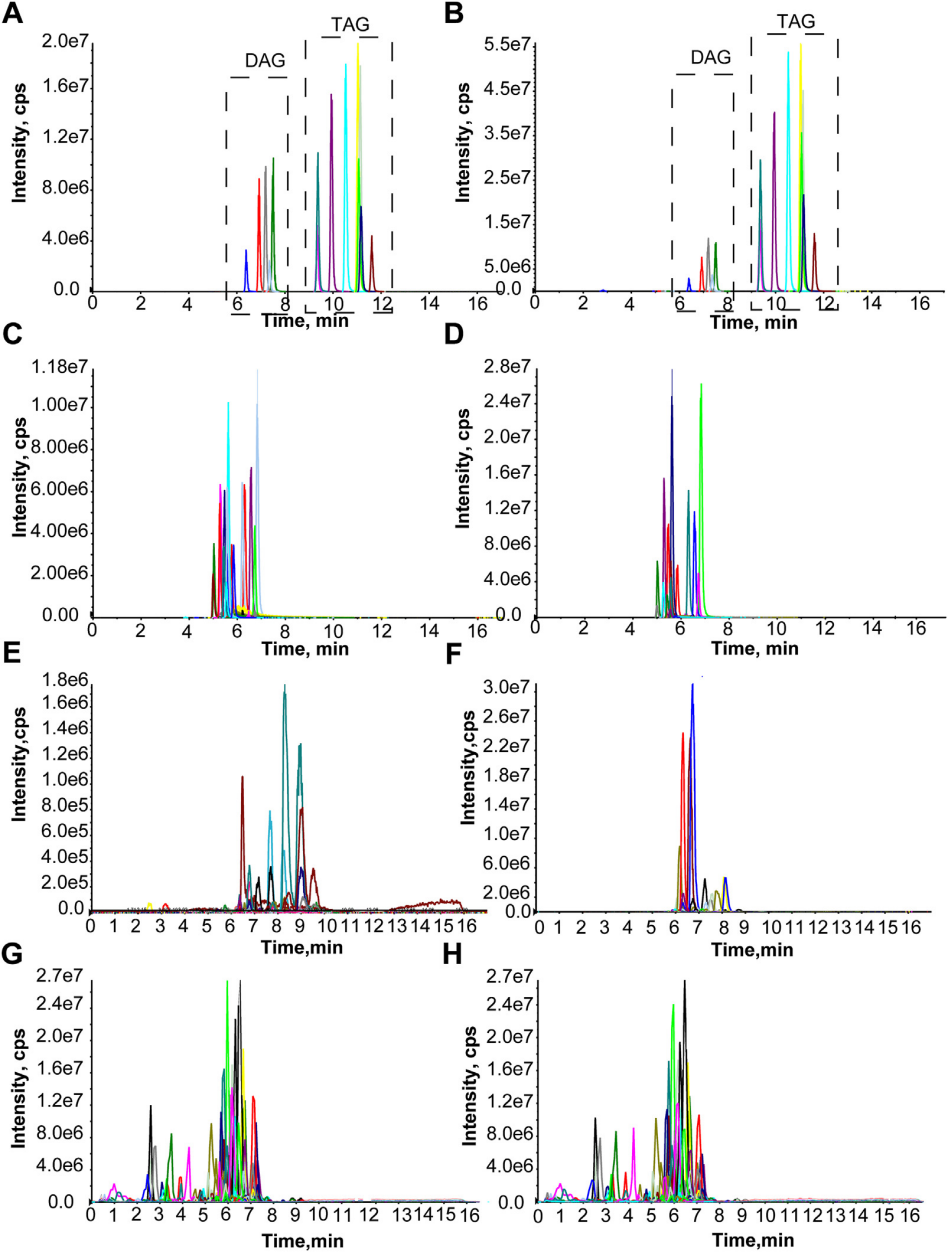
Multiple MRM ion pair validation using ISs and sample extracts. Different MRM ion pairs of DAG and TAG (A and B) and phospholipids (C and D) from ISs were detected in positive and negative ESI mode, respectively; Sample extracts were detected using two different MRM ion pairs of DAG (E and F) and phospholipids (G and H) from odd-chain fatty acyl lipid library in positive and negative ESI mode, respectively.

**Fig. 3:**
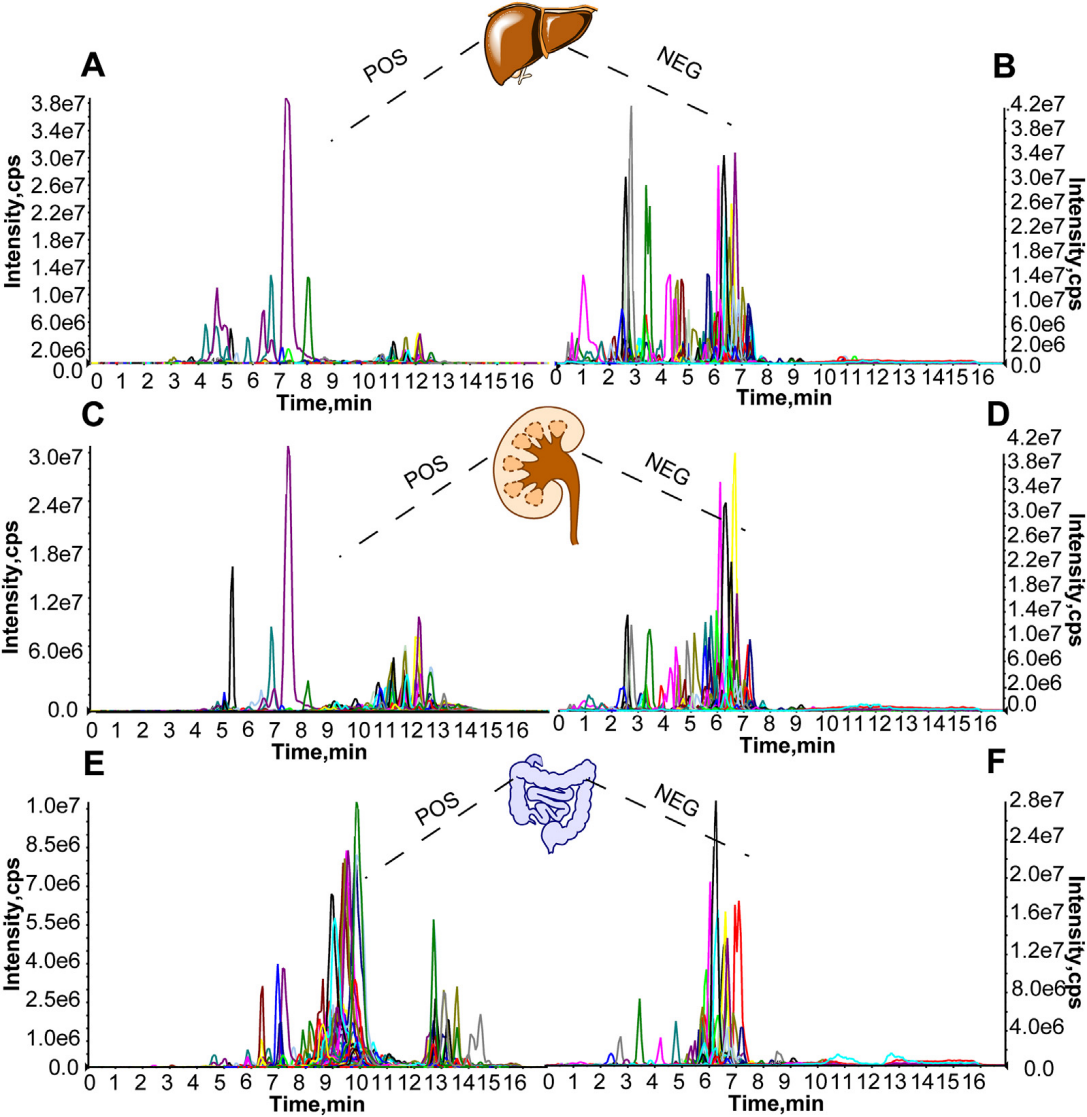
The existence of odd-chain fatty acyl lipids in various mouse tissues. Odd-chain fatty acyl lipids were detected in mouse liver (A and B), kidney (C and D), and intestine (E and F) tissue extracts in both positive and negative ESI mode, respectively, x represents RT (min), y represents intensity (cps).

**Fig. 4:**
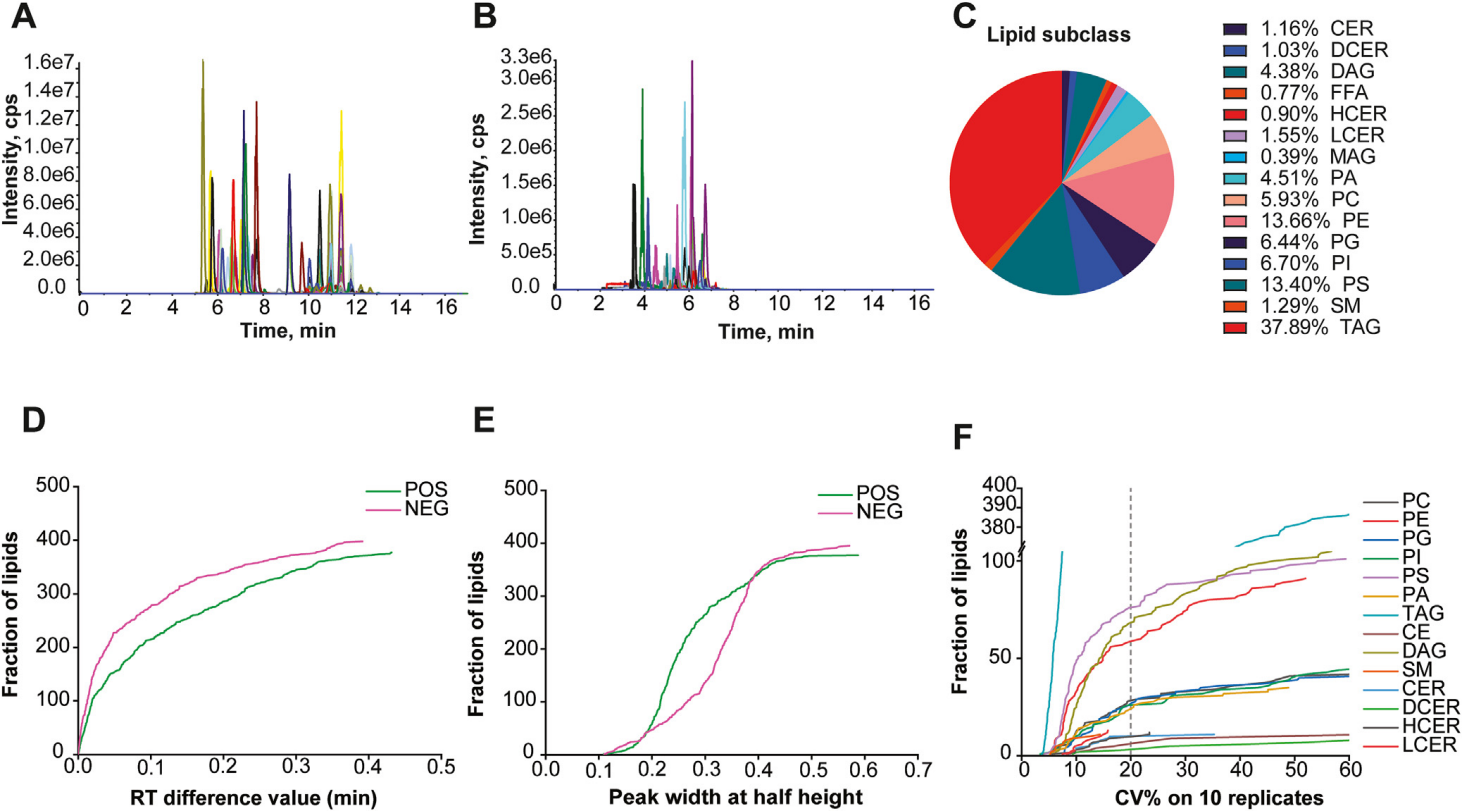
Odd-chain fatty acyl lipidome and robustness analysis. TIC figure of clinical colon cancer tissue extracts detected in positive (A) and negative (B) ESI mode, x represents RT (min), y represents intensity (cps); different subclass of lipids detected in the method and its fraction (C); RT difference between the predictive and real detective value in both positive and negative ESI mode, x represents RT difference value (min), y represents accumulated frequency of lipids (D); peak width at half height, x represents RT (min), y represents accumulated frequency of lipids (E) ; CV% statistics of lipid subclasses on 10 consecutive injection, x represents CV(%), y represents accumulated frequency of lipids (F).

Other statistical analyses were performed using the two-tailed unpaired Student’s *t-*test or one-way ANOVA with Dunnett multiple comparison test as indicated in GraphPad Prism, version 9.0.0 (GraphPad Software, Inc). *P* values (∗∗∗*P* < 0.001, ∗∗*P* < 0.01, and ∗*P* < 0.05) were autolabeled in the figure. Line graphs in Fig. 1, Fig. 2, Fig. 3, Fig. 4 were plotted by OriginPro 2021 (64 bit) 9.8.0.200. The Adobe Illustrator CS4 was applied to process graphics and prepare final figures.

## Results

### Targeted MRM ion pair building based on ECN intraclass RT prediction model

We first created a library of odd-chain acyl lipid library based on LIPID MAPS and in silico modeling. The lipid library could be downloaded directly from LIPID MAPS Structure Database (https://www.lipidmaps.org/databases/lmsd/browse). Lipid checking and integrating was performed by choosing acyl containing FA13:0, FA15:0, FA17:0, FA19:0, FA21:0, and FA23:0 moieties. Monounsaturated and diunsaturated acyl of those mentioned moieties were generated simultaneously via 2 or 4 Da mass defect *in silicon*. In total, a library containing FAs, glycerolipids, glycerophospholipids, and sphingolipids was preliminarily generated. This provided exact molecular weight information of certain lipids for subsequent targeted MRM ion transition setting.

Although TAGs are normally termed as nonpolar lipids or neutral lipids, pseudo molecular ion of TAG can be formed with ammonium, this firmly increase the signal response in positive ESI mode, and thus the alteration in the acyl chain length and the degree of unsaturation determine the ionization efficiency of different TAG molecular species (27, 28). Accordingly, the neutral loss of 259, 287, and 315 Da was assigned to TAG containing FA15:0, FA17:0, and FA19:0 moiety, respectively (Fig. 1A). In contrast to TAG, diacylglycerols (DAGs) have a free hydroxyl group and only two fatty acyl residues, which led them easier to construct daughter ion of *m*/*z* 299, 327, and 355 Da for FA15:0, FA17:0, and FA19:0 moiety contained DAG, respectively (Fig. 1A). In addition, if the fatty acyl chain contains one, two, or more double bonds, the mass defect will be 2, 4, or the number of bond multiplied by 2 Da with its corresponding saturated lipid species. This was also consistent with other lipids in collision-induced dissociation behavior during ionization, such as DAG, SM, ceramides, etc. (Fig. 1A).

MRM ion transitions for glycerophospholipids were then generated based on their general mass fragmentation patterns (29, 30), in which differing both in their combinations of fatty acyl residues attached to either the sn-1 or sn-2 positions of the molecule and in the chemical linkage of the polar head group to the sn-3 position of the glycerol backbone were considered. Under negative ESI mode, the collision-induced dissociation of the carboxylate anions from phosphatidic acid (PA), phosphatidylcholine (PC), phosphatidylethanolamine (PE), phosphatidylglycerol (PG), phosphatidylserine (PS), phosphatidylinositol (PI) containing the odd-chain fatty acyl residues was deduced (Fig. 1B). Consequently, this led to two fragmentation modes for each phospholipid where fatty acyl was located either at sn-1 or sn-2 site. ISs were exploited subsequently for further validation, and both ion pairs for each individual lipid shared strong signal response in total ion chromatograph (TIC) under optimized ESI detection condition, prompted us to use either odd chain directly or the other fatty acyl residues as the detection ion paring with molecular ion in MRM transitions. Moreover, the abundance ratios of the sn-1 to sn-2 carboxylate anions determined were dependent on the collision energy, fatty acyl chain length, and degree of unsaturation, which varies greatly among differential lipids. This reminded us to take these potential impact factors into consideration when it comes to the identification of odd-chain fatty acyl lipids as there were only limited number of internal or authentic standards. To encounter these challenges, IS belonging to the same lipid subclass was assigned to lipid with identical number of chain length and double bonds. The collision energy, decluttering potential, and dwell time of ISs in ion trap were reciprocally used as reference values for targeted lipids.

Using the above fragmentation rules of lipids, we next produced the MRM ion transitions of UltimateSPLASH™ ONE ISs for deep confirmation. With the optimized LC elution program, we successfully achieved the TIC of these deuterated standards in both positive and negative ESI mode (Fig. 1C, D and supplemental Table S1), which suggested the feasibility of corroborating the other odd-chain fatty acyl-contained lipids in the established lipid library.

Continually, lipid RT in reverse LC is becoming increasingly common to supplement valuable information to lipid identification, termed the ECN model. This model enables the prediction of RT of lipids with the same head group and describes the effects of acyl chain length and degree of saturation on RT (31, 32, 33). Generally, the second-degree polynomial regression curves are fitted according to lipid relative dependencies of RTs on the number of carbon or double bonds (34). In line with this, we initially performed binomial fitting via RT of ISs, as shown in Figures 1E, F, S1, and supplemental Table S10, the regression lines displayed good linearity with correlation coefficients (*R*^2^) of 0.97 or above, and the link between reduced RT with added unsaturation or shortened carbon chain length and vice versa. Albeit partially, this model brought a majority of lipids in our library to gain another reference index for lipid identification. The fit equation of other lipid subclasses, for example, PA, was deduced by using high signal/noise response peaks of lipid because of a lack of corresponding deuterated ISs in the kit.

### Lipid validation via multi-MRM transitions, deuterated ISs, and authentic standards

The above established model using multidimensional match including molecular ion, product ion, dissociation behavior, and RT provides multiple structural information and helps to fulfill lipid high-coverage identification. Further validation was performed using different MRM transitions based upon various fatty acyl residues of ISs such as three MRM transitions for TAG (Fig. 2A, B), two transitions for DAG (Fig. 2A, B), and phospholipids (Fig. 2C, D). Strong signal response of MRM transitions was evidenced experimentally other than differing in peak intensity. This was also approved by the sample extracts as multi-MRM transition of DAG (Fig. 2E, F) and phospholipids (Fig. 2G, H) exhibited similar TIC spectra. Considering TAG contains three fatty acyl residues, we generated a combination of TAG with various MRM transitions, as shown in supplemental Fig. S2A–D, each TAG isomer displayed nearly the same RT, this complicated the identification of certain chain length and double bonds in fatty acyl residues as exemplified by TAG51:3 (supplemental Fig. S2D), which have the possibility of 15:0-15:1-21:2 TAG, 15:0-17:0-19:3 TAG, 15:0-17:1-19:2 TAG, 15:0-17:2-19:1 TAG, 15:0-19:0-17:3 TAG, etc.

To solve this problem, we tentatively generated a combination of fatty acyl residues of each TAG and verified the aliphatic chains in LC-MS/MS using a series of lipid extracts from mice tissues including heart, liver, lung, kidney, and adipose. As a result, a total of 45 TAGs were identified with certain fatty acyl chains (supplemental Figs. S3–S5). Considering differentiated signal response of various fatty acyl in sn-1, sn-2, or sn-3, our results only showed the possibility of certain fatty acyl residues other than their respective location in TAG scaffold. In addition, DAGs were also conformed through two MRM transitions, and a number of 60 DAGs were assigned through their signal response and RT. In negative ESI mode, a total of 204 phospholipids were validated with the identical RT for both MRM transitions. Among which, PA (17:0/18:1) (supplemental Fig. S6A), PE (17:0/16:1) (supplemental Fig. S6B), PG (17:0/18:1) (supplemental Fig. S6C), PI (17:0/16:1) (supplemental Fig. S6D), PI (17:0/18:1) (supplemental Fig. S6E), and PS (17:0/18:1) (supplemental Fig. S6F) were confirmed with two MRM transitions. Moreover, the peaks of deuterated ISs for these lipids except PA (17:0/18:1) were also demonstrated simultaneously in the same run.

We next used biological sample extracts to verify these lipids through comparing with commercial available and a limited number of authentic standards or deuterated ISs. Authentic standards of TAG45:0-FA15:0 and TAG51:0-FA17:0 nearly overlapped with themselves in samples (supplemental Fig. S2E, F). TAG43:1-FA15:0 (supplemental Fig. S7A), TAG49:1-FA15:0 (supplemental Fig. S7B), TAG51:2-FA17:1 (supplemental Fig. S7C), TAG53:3-FA17:0 (supplemental Fig. S7D) in positive ESI mode, LPC (17:0) (supplemental Fig. S7E), LPE (17:0) (supplemental Fig. S7F), PE (17:0/16:1) (supplemental Fig. S7G), PG (17:0/18:1) (supplemental Fig. S7H), PI (17:0/16:1) (supplemental Fig. S7I), and PS (17:0/16:1) (supplemental Fig. S7J) in negative ESI mode both matched excellently with their own ISs in TIC spectra. These results illustrated the similar RT of lipids with their own ISs, boosting the confidence of odd-chain fatty acyl lipid identification.

Thus far, a total of 309 odd-chain fatty acyl lipids (supplemental Table S11) were conformed either by MRMs or a limited number of standards. Nevertheless, the exact structural identification of these lipids has to resort to extra high-resolution mass spectral fragmentation by which odd-chain fatty acyl lipids may lead to the ionization suppression as low abundance. Hence, the ECN-based RT prediction or MRM transitions could be a useful supplementary to analyze these lipids.

### The existence of odd-chain fatty acyl lipids and their isomers

To exclude the false-positive results and corroborate the existence of these odd-chain fatty acyl lipids, we transferred our established odd-chain fatty acyl lipid library to another Exion UHPLC-AB SCIEX 6500+ platform. Mouse tissue extracts from liver (Fig. 3A, B), kidney (Fig. 3C, D), and intestines (Fig. 3E, F) displayed significant peak response in both positive and negative ESI mode, respectively, illustrating the existence of these rare lipids.

In addition, the existence of isomers complicates the lipid structural identification. This lies in varying in the location of fatty acyl region- and stereoisomers or (*cis*/*trans*) geometry of carbon-carbon double bonds. We noticed a set of lipids with isomers through RT variation in reverse LC, such as TAG47:1, TAG49:1, TAG51:2, and TAG51:3 in positive ESI mode (supplemental Fig. S2A–D). Notably, phospholipids in negative ESI mode also exhibited doublet peaks including PC (21:0/18:1), PE (21:0/18:1), PG (17:1/18:1), PA (17:0/18:1), PI (17:0/18:1), and PS (17:0/18:1) (supplemental Fig. S8A–F).

Even though the origin, metabolism, and biological function of these lipids were largely unknown, the above qualitative results enhance our understanding of these lipids, and the presence of odd-chain fatty acyl lipid isomers lay the experimental foundation for further structural identification and quantitative analysis.

### Odd-chain fatty acyl lipidome and robustness analysis

By applying the built lipid library and its MRM transitions with optimized LC elution condition to prepared clinical colon cancer tissue extracts, we aimed at lipids with positive signal response (signal/noise 10 or above) in both positive or negative ESI mode. Afterward, a targeted-MS-based lipidomic screening method using a combination of variable RT window width was proposed, which was specific for each lipid and could increase the throughput through reducing the analysis time of a sMRM method (35). Likewise, the quality of the peaks can be improved apparently without compromising the cycle time by varying dwell time for each transition. Synchronically, allocating a short dwell time to lipids with high abundance or vice versa speeds up accommodating large number of MRM transitions in a single run with improved data quality.

Using this method, we determined the total ion chromatogram of sample extracts as shown in Figure 4A, B in positive and negative ESI mode, respectively, sharing good peak intensity and separation. A number of 776 lipid species with odd-chain fatty acyl residues were accumulated. Among which, phospholipids containing PA, PC, PE, PG, PI, and PS as a whole have the largest proportion of 50.64%. Besides, a percentage of 37.89% for TAG was the most abundant single subclass of all lipids, whereas monoacylglyceride only shared a minimum part of 0.39%. Ceramide, dihydroceramide, hexosylceramide, lactosylceramide, and free FA (FFA) were comparably closed to 1%, which was about a quarter of DAG at 4.38% (Fig. 4C).

The robustness of the method was next evaluated via RT difference between the predictive and detected value. As shown in Figure 4D, the majority of the RT defect values lied at 0.2 min, even the largest RT defects were smaller than 0.5 min in both positive and negative ESI modes, indicating the brilliant capacity of the ECN-based RT prediction model. Peak width at half height was further deployed as shown in Figure 4E, the shape of peaks in the positive ESI mode possessed relatively better performance than that of lipids in negative mode, even if the peak widths in both modes were fluctuated from 0.1 to 0.7 min, showing the excellent lipid separation during LC elution process. Correspondingly, the reproducibility of the method was ascertained by determining CV% in 10 consecutive injection of prepared sample extracts. As results shown in Figure 4F, the main body of lipid subclasses had CV% within 20% accompanied by an even less than half of that value for TAG. The peak area fluctuation of deuterated ISs through 10 consecutive injections in positive and negative ESI modes was also substantiated by the stability of the method (supplemental Fig. S9A, B). Together, these results indicate the good robustness and reproducibility of the developed method.

### Odd-chain fatty acyl lipidomic profiling of tissues from clinical colon cancer reveals altered triglyceride

**Fig. 5:**
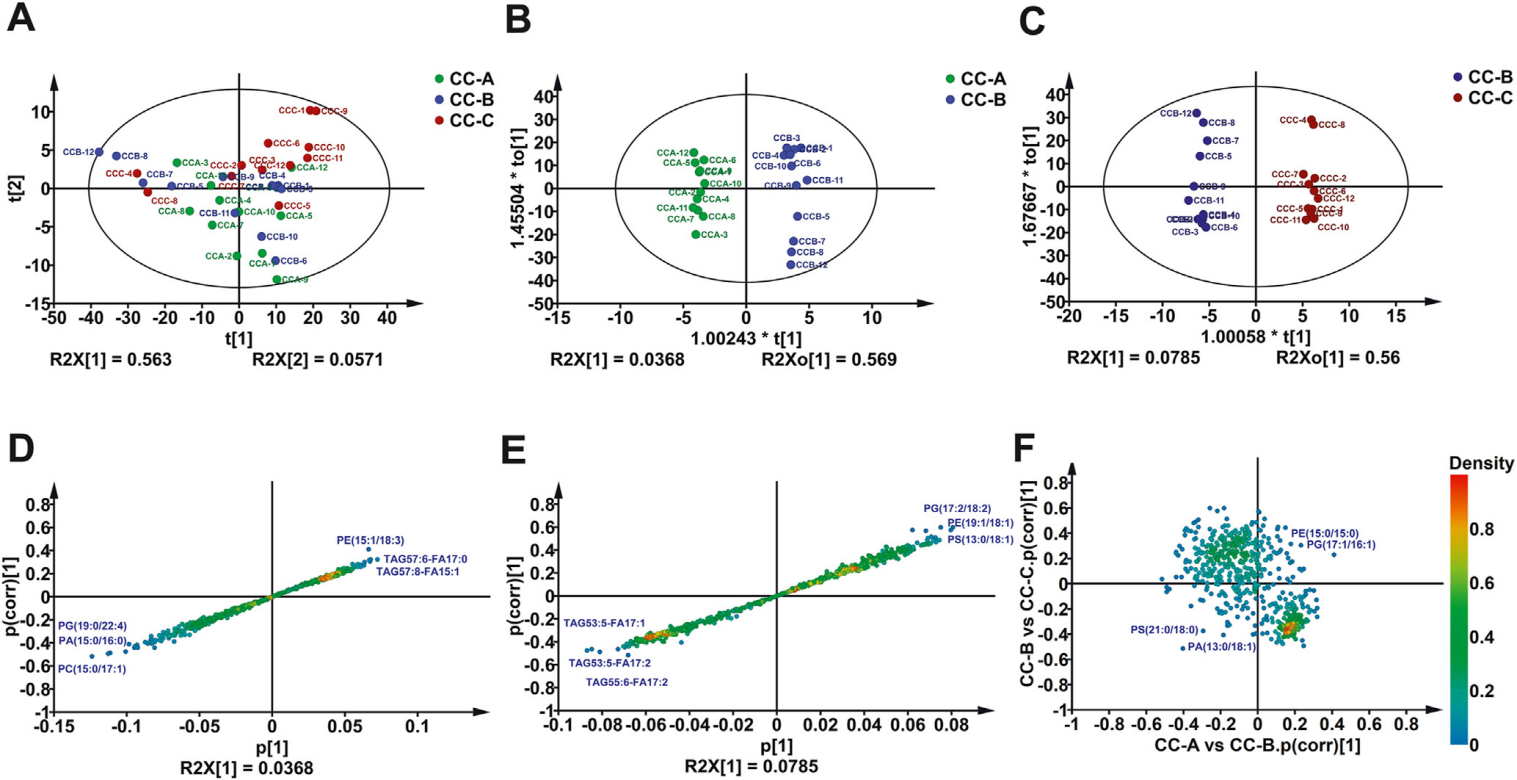
Odd-chain fatty acyl lipidomic analysis of clinical colon cancer. PCA analysis of whole samples (A), CC-A in green, CC-B in blue and CC-C in red; OPLS-DA analysis of CC-A versus CC-B (B); OPLS-DA analysis of CC-B versus CC-C (C); S-plot analysis of CC-A versus CC-B (D); S-plot analysis of CC-B versus CC-C (E); SUS-plot analysis between CC-A versus CC-B and CC-B versus CC-C (F). Color density represents the distribution of lipids.

We next investigated the dynamics of odd-chain fatty acyl lipids during the progression of colon cancer through the above method. The nonmalignant tissues including distant normal (namely CC-A in green) and adjacent tissues (namely CC-B in blue) and matched malignant tissues (namely CC-C in red) of 12 descending colon cancer patients were used for analysis. A PCA of data dispersion of each group based upon the lipidomic profile showed no considerable outliers and only minor group differentiation (Fig. 5A), followed by OPLS-DA displayed clear group clustering of CC-A and CC-B (Fig. 5B), CC-B and CC-C (Fig. 5C), respectively. The lipid species with the highest abundance differences between case and control samples were visualized by S-plots (Fig. 5D–E, whereby lipid abundance downregulated in samples are marked in blue, and upregulated lipid species are marked in red). TAGs were remarkably upregulated comparing lipid abundance ratio of CC-B versus CC-A, whereas phospholipids such as PG, PA, and PC, were significantly downregulated (Fig. 5D). By contrast, an inverse trend was found in CC-C/CC-B (Fig. 5E). The two OPLS-DA models were then correlated by the Shared and Unique Structures Plot to identify appropriate variables for the construction of disease biomarkers in the form of multivariate predictive scores. A mainstream of lipids distributed in the second (phospholipids) and fourth (TAG) quadrant of the coordinate represents the uniquely fluctuated lipids. While the first and third quadrants of the coordinate are dispersed by a majority of phospholipids, suggesting the shared characteristics of lipids with simultaneously increased or decreased abundance, respectively (Fig. 5F).

**Fig. 6:**
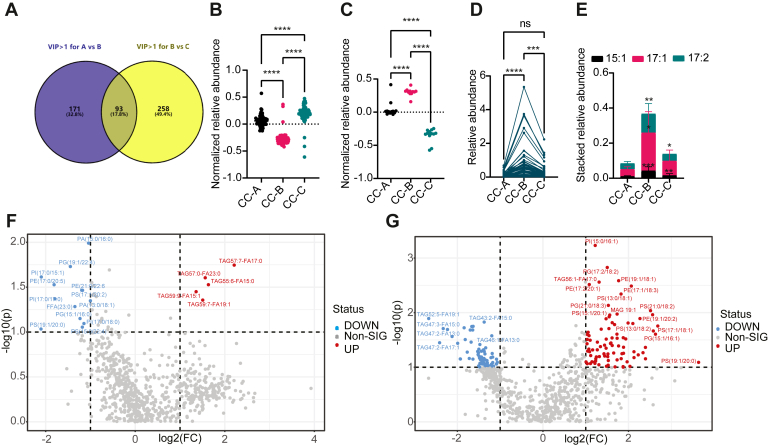
Odd-chain fatty acyl lipidomic analysis of clinical colon cancer. Venn diagram of lipids with VIP >1 between CC-A versus CC-B (in blue) and CC-B versus CC-C (in yellow) through OPLS-DA analysis (A); statistic analysis of the relative abundance of phospholipids with VIP >1 in 93 intersected lipids in A (B); statistic analysis of the relative abundance of TAG with VIP >1 in 93 intersected lipids in A (C); the relative abundance of TAG in three groups (D); the stacked relative abundance of lipids containing FA15:1, 17:1, and 17:2 residues in three groups (E); Volcano analysis of CC-A versus CC-B (F); Volcano analysis of CC-B versus CC-C (G). Downregulated lipids were in blue, and upregulated lipids were in red.

Furthermore, VIP values >1 were generally defined as statistically relevant and lipids with VIP >1 in both OPLS-DA models (CC-A vs. CC-B and CC-B vs. CC-C) were intersected in the Venn diagram with 93 types of lipids (Fig. 6A). Among which, the notably decayed abundance of odd-chain fatty acyl phospholipids and preferentially accumulated abundance of odd-chain fatty acyl TAG in CC-B were evidenced by comparing with either CC-A or CC-C (Fig. 6B, C). Regardless, the variation of TAG abundance may be responsible for the lipids containing FA15:1. FA17:1 or FA17:2 moieties in positive ESI mode as TAG with VIP >1 or TAG were more abundant in CC-B (Figs. 6C–E and S10). Whereas, there was no noteworthy shift of phospholipids with VIP >1 or phospholipids with different fatty acyl residues alone or together with six subtypes of phospholipids, including PA, PC, PE, PG, PI, and PS (supplemental Fig. S11).

Volcano plot combines results from FC analysis and *t*-tests into one single graph, which can be used to intuitively select significant features based on either biological significance, statistical significance, or both. Based on this, we performed volcano plot analysis with FC ≥2 and *P* value <0.1 as especially and statistically significant for the lipidomic differentiation, such as selected phospholipids and TAG in red and blue (Fig. 6F, G). The volcano plot showed upregulated TAG including TAG57:7-FA17:0, TAG57:0-FA23:0, TAG59:9-FA15:1, TAG59:7-FA19:1, and TAG55:6-FA15:0 in CC-B/CC-A coinciding with downregulated TAG52:5-FA19:1, TAG43:2-FA15:0, TAG47:3-FA15:0, TAG47:2-FA13:0, TAG47:2-FA17:1, and TAG46:1-FA13:0 in CC-C comparing to CC-B. Concurrently, phospholipids, such as PA (15:0/16:0), PI (17:0/15:1), PE (17:0/20:5), PI (17:0/17:0), PS (19:1/20:0), were evidently reduced in CC-B/CC-A but PI (15:0/16:1), PE (19:1/18:1), PS (21:0/18:2), PS (17:1/18:1), PG(15:1/16:1) were enhanced remarkably in CC-C versus CC-B (Fig. 6F, G).

Meanwhile, the validated lipids using MRM transitions were also sorted from the above quantitative results and found that a number of 237 validated and odd-chain fatty acyl lipids (supplemental Table S12) were quantified from these colon cancer tissues. Subsequent analysis by using MetaboAnalyst showed that the OPLS-DA results of CC-A versus CC-B (supplemental Fig. S12A) and CC-B versus CC-C (supplemental Fig. S12B) were similar to the following results. The decay of odd-chain fatty acyl TAGs in CC-C was also observed in the S-plots (supplemental Fig. S12C, D) and heat map (supplemental Fig. S12E).

Consequently, considering statistical parameters (FC, *P* value, and VIP) and excluding exogenous interference, the lipid species TAG were of the highest relevance for the differentiation with the progression of colon cancer.

### Remodeling of odd-chain fatty acyl lipid profile is associated with deregulated metabolic gene transcription and protein expression in colon cancer

In light of the above findings with standard clinical parameters for colon cancer, we determined the serum lipid profile of patients, but there was no obvious association between odd-chain fatty acyl contained TAG with serum total TAG concentration, as their serum concentration of TAG or other lipoproteins showed no noticeable fluctuation (supplemental Tables S2 and S3).

We further analyzed the level of odd-chain FFA because exogenously dietary fats are the major source of these lipids. Both total and each individual odd-chain FFA levels were descending in the order of normal, adjacent, and tumor tissues (supplemental Fig. S13), implying the beneficial effects approved by accumulating epidemiological studies (21).

**Fig. 7:**
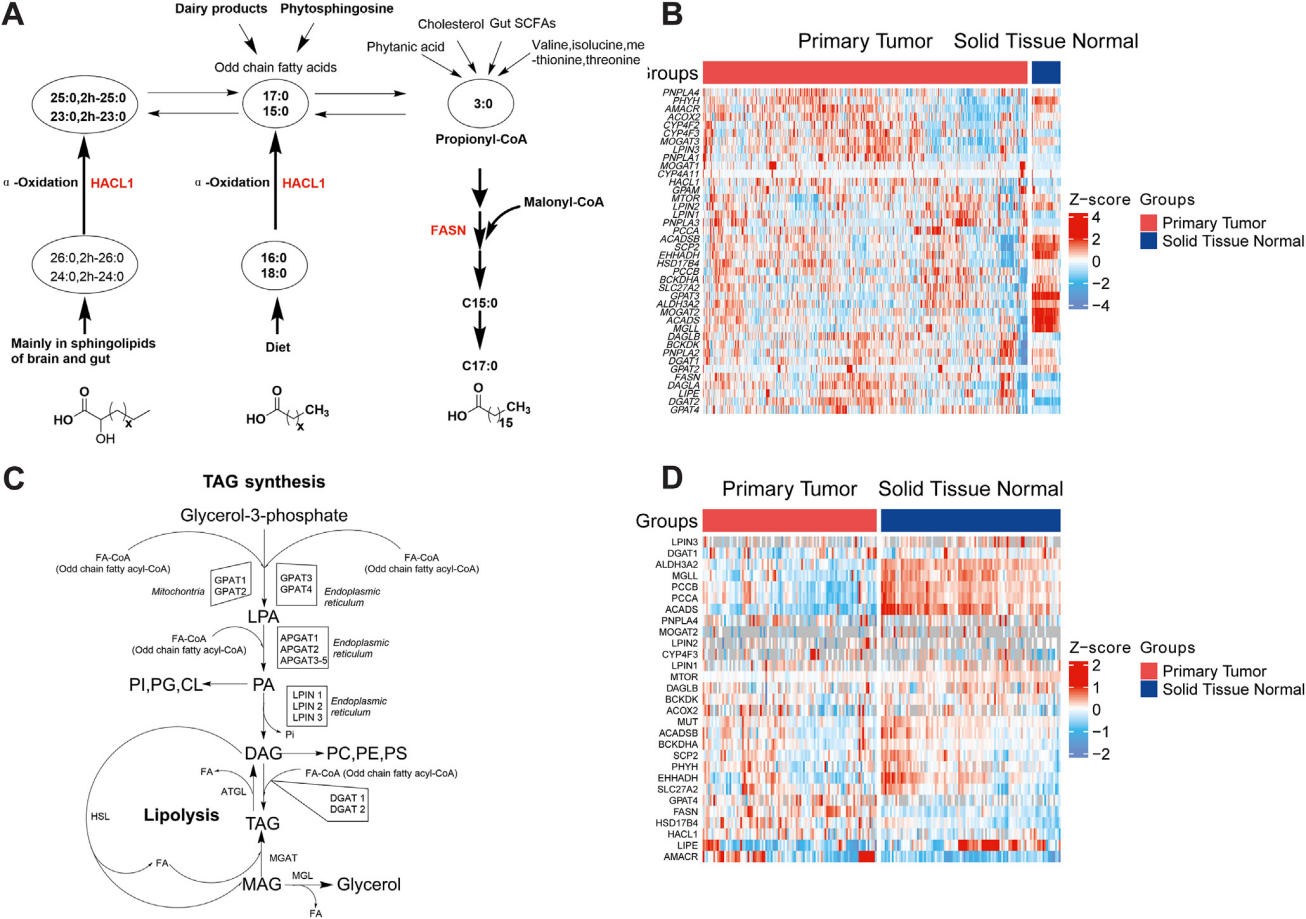
Dysregulated lipid metabolism in colon cancer. OCFA metabolism pathway as illustrated by key enzymes (A); the RNA-Seq profile of genes involving OCFA metabolism in colon cancer from TCGA (B); TAG metabolism pathway as illustrated by key enzymes (C); proteomic profile of enzymes involving OCFA metabolism in colon cancer from CPTAC (D). Color gradient of Z-score shows the expression level of mRNA or protein.

We then diverted to the gene transcription of odd-chain fatty acyl lipid metabolism to link lipids with genomic and proteomic features of colon cancer. Serendipitously, key genes involving the catabolism of BCAA, which produce propionic acid (C3:0) (Fig. 7A) for odd-chain FA endogenous biosynthesis and transformation, were downregulated significantly such as *ACADS*, *ACADSB*, and *BCKDHA* co-occurred with markedly enhanced *BCKDK* expression in colon cancer through The Cancer Genome Atlas database (Fig. 7B). A significantly lower abundance of SCFAs including propionate and SCFA-producing bacteria has also been demonstrated in CRC (36), representing reduced primer for odd fatty acyl elongation and complex lipid synthesis despite of relatively high expression of *FASN* (Fig. 7A, B) and upregulated TAG synthesis (Fig. 7B, C). PCCA and PCCB are the key enzymes in charge of transforming propionate to methyl malonic acid, both of which showed lower expression (Fig. 7A, B), but accumulated propionate was not observed in colon cancer, suggesting that the diminished propionate originated from upstream metabolic transfer played a primary role for the abundance. Moreover, key genes of peroxisomal α-oxidation of long-chain FAs like *HACL1* and *PHYH* obscure the transformation as paradoxical gene expression was observed (Fig. 7B). Yet, metabolic flux experiment using [U-^13^C] valine, [U-^13^C] isoleucine, [U-^13^C] threonine, and [U-^13^C] methionine consolidated the above hypothesis that BCAA catabolisms are responsible for the production of propionate (37). Protein expression level of these targeted metabolic genes was further corroborated by the gene transcription (Fig. 7D). Thus, the compromised OCFA as scaffold was marginally incorporated into TAG following the synthesis pathway (Fig. 7C).

Together, the decreased odd-chain FFA largely contributed to the altered TAG, but genomic and proteomic aberration in BCAA catabolism may further exacerbate the OCFA for TAG synthesis endogenously during clinical colon cancer.

## Discussion

In this study, we proposed a targeted odd-chain fatty acyl-containing lipidomic analysis method based on ECN intraclass RT prediction. Further lipid identification in sample extracts matching with deuterated ISs and authentic standards enhanced the fidelity of the established processes. Among which, lipid isomers extensively distributed in several subclasses of lipids as differing in carbon number and linking site of carbon-carbon double bonds, this diversify the structural information of the lipids. Through validation processes, the method displayed good reproducibility and robustness as shown by peak width at half height within 0.7 min and CV% under 20%. In total, a number of 776 lipid species with odd-chain fatty acyl residues could be detected in ESI mode, representing the high-throughput lipid screening ability of the method.

Using reverse LC-MS-targeted and sMRM lipidomics to sensitively quantify odd-chain fatty acyl-contained lipids in clinical colon cancer tissues, we provided new insight into the noticeably lowered TAG in tumor tissues compared with the adjacent normal tissues, underlying the beneficial effect of dietary OCFAs. Accumulating studies have shown that OCFAs (mainly C15:0 and C17:0) are inversely associated with a range of diseases, including type 2 diabetes mellitus (24), coronary heart disease (38), CRC (21, 39, 40), etc., suggesting that dietary OCFAs could boost health and reduce disease risk. Clinical cohort studies further validated these results (21, 24, 39, 40). Yet, whether boosting dietary odd-chain fatty acyl lipids benefits the therapeutic efficacy of colon cancer patients needs further corroboration.

The novel changes in odd-chain fatty acyl-containing TAG switched in this study may be related to reported alterations in enzymes involved in lipid metabolic reprogramming. Tumor-specific activation/inactivation patterns of these individual enzymes, most likely driven by tumor-specific oncogenic signaling, may lead to unique TAG profile that is a key characteristic for tumors. Even subtle changes within the lipidome may be critical to support the cancer phenotype and treatment resistance. Therefore, it is likely to be more clinically potential to precisely target the enzymes and pathways and thus reverse the remodeling of altered lipids.

In addition, previous studies about lipidomics largely refer to even-chain fatty acyl lipids, which helps to further shape a stubborn image of research pattern attached to even carbon chain number naturally. Several studies used odd-chain fatty acyl-containing lipids as quantitative ISs (9). Among which, C15:0 and C17:0 FAs were the most widely employed. As the importance of odd-chain fatty acyl lipids is increasingly noticed, they should be added to the routine lipidomic analyses. To our knowledge, our study is the first report of odd-chain fatty acyl-related changes in lipidomic profiles and the association of lipid metabolic genes and enzymes in clinical colon cancer.

Nonetheless, the limitations of our study should be taken into account. First, the lipid library in this study only included odd-chain fatty acyl residues, neither fatty ether nor fatty vinyl ether were considered, the process of structural identification and RT prediction of lipids were simplified. Whereby, the diversity of lipids should also be included in depth. Moreover, QTRAP MS was utilized in the study as its excellent quantitative performance, but a lack of fragmentation ions in high resolution made it impossible to produce enough information about collision-induced dissociation. Therefore, the ECN-based RT prediction combined with internal or authentic standards validation was a trade-off strategy, future studies should exploit more reference index for cross validation of lipid profile. Eventually, our findings warrant further experiments using machine learning-based training and validation set in enlarged cohort and functional investigation of lipidome in other cancer types, to unravel the molecular mechanisms underlying these changes and to explore the impact on metabolic functioning and, ultimately, on cancer progression.

In conclusion, we developed a reproducible, robust, and high-throughput lipidomic profiling approach for the detection of clinical colon cancer tissues, which is applicable for the screening of odd-chain fatty acyl-containing lipids during the pathogenesis of cancer. Thus, odd-chain fatty acyl lipidomic profiling of colon biopsies, possibly used as meaningful supplementary to recent clinical total TAG quantification, has the potential to provide new information about disease features and, potentially, patient responsiveness to therapeutics.

## Data Availability

All data supporting this study are included in the article and supplemental data.

## Supplemental data

This article contains supplemental data.

## Conflict of interest

The authors declare that they have no conflicts of interest with the contents of this article.
