# Supplementary figures and images for: Equivalent carbon number-based targeted odd-chain fatty acyl lipidomics reveals triacylglycerol profiling in clinical colon cancer
Source: J Lipid Res. 2023 May 29;64(7):100393. doi: 10.1016/j.jlr.2023.100393 (PMC10331287; doi:10.1016/j.jlr.2023.100393)

**A**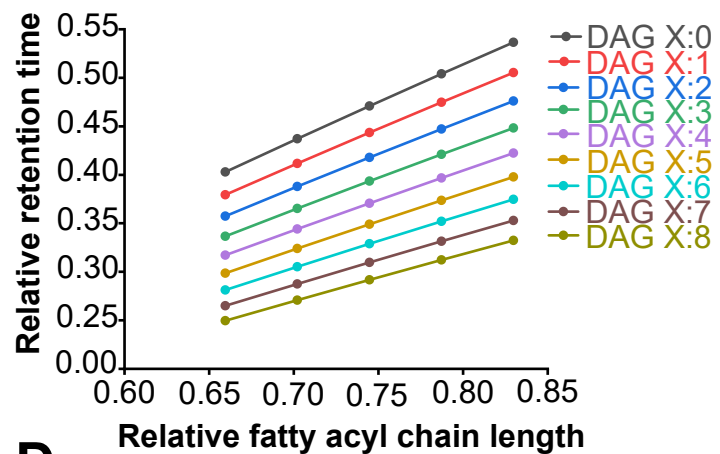**B**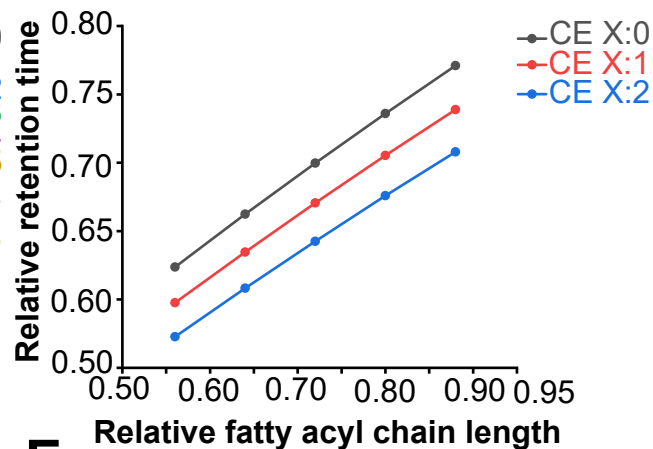**C**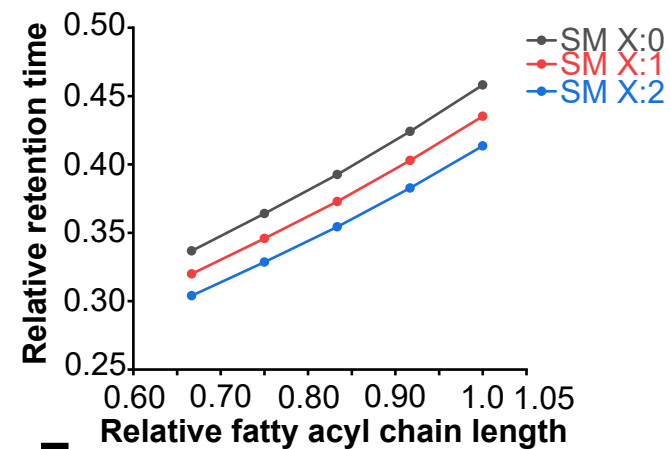**D**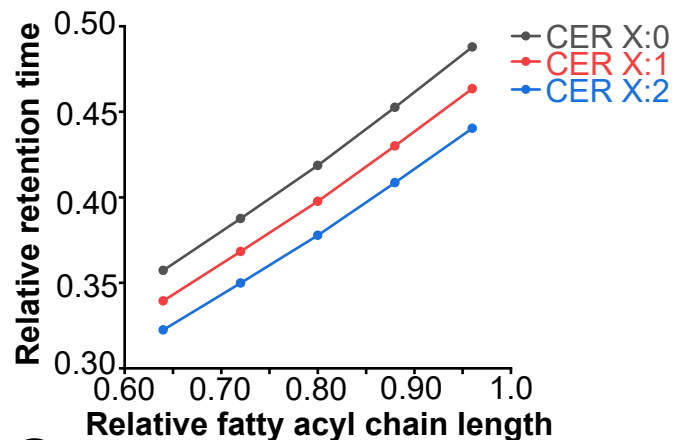**E**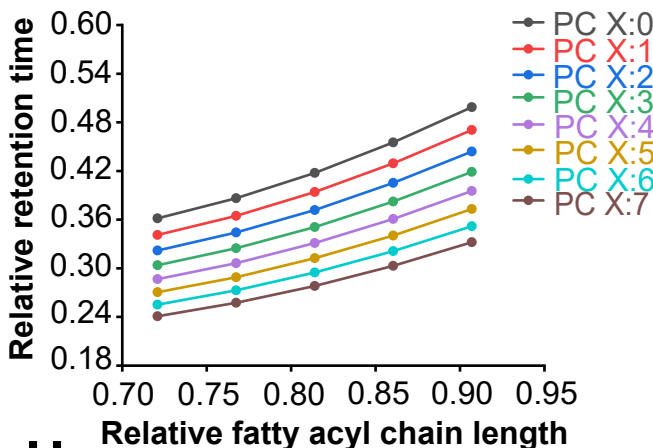**F**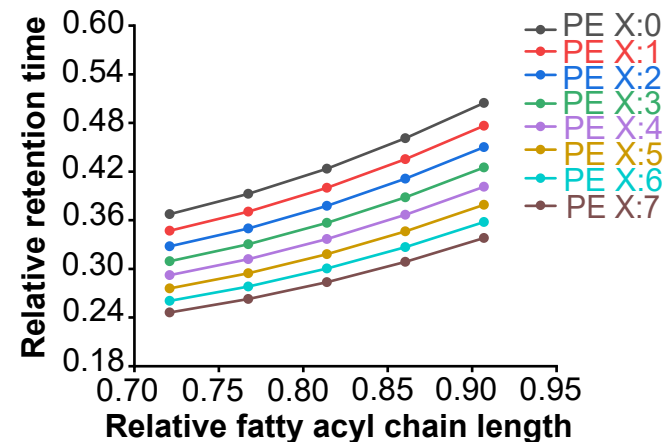**G**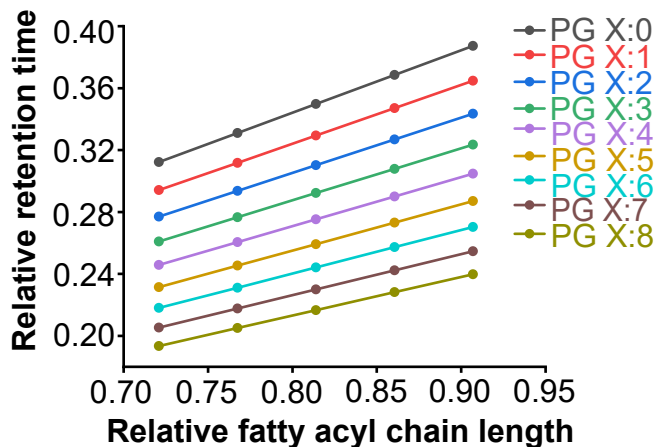**H**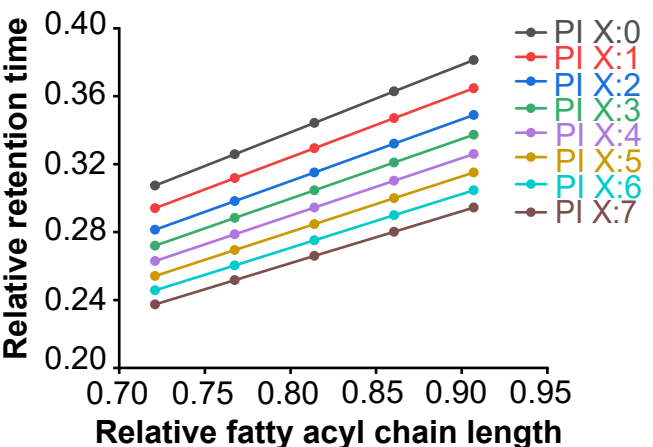**I**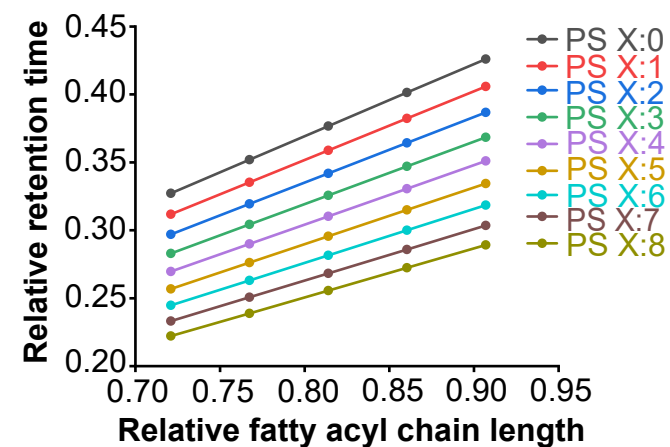

Supplement: Fig S1 [file mmc1.pdf]

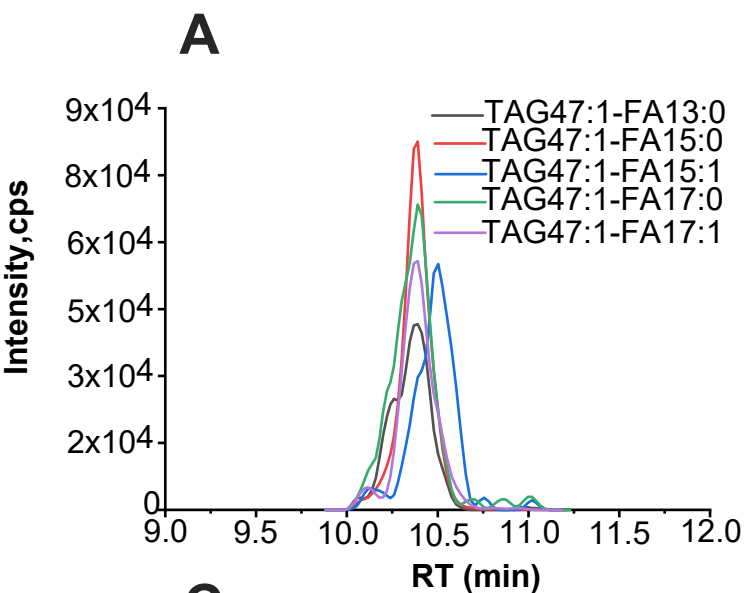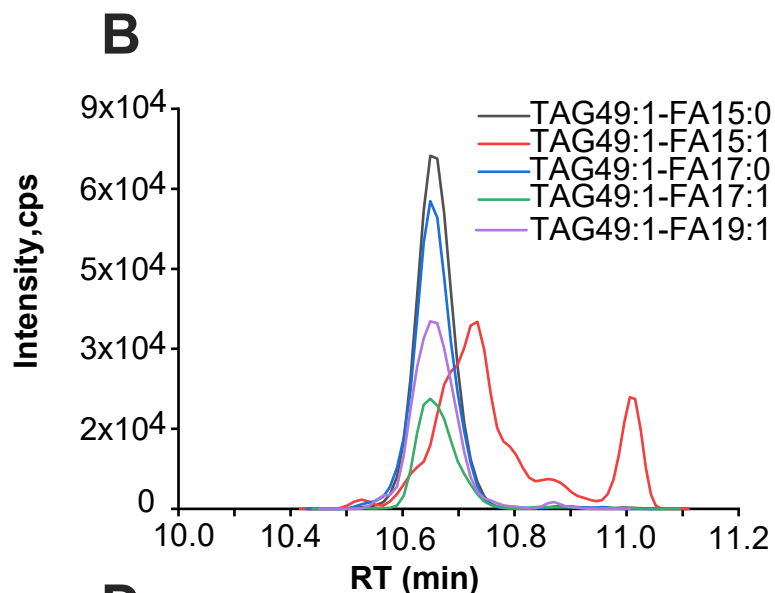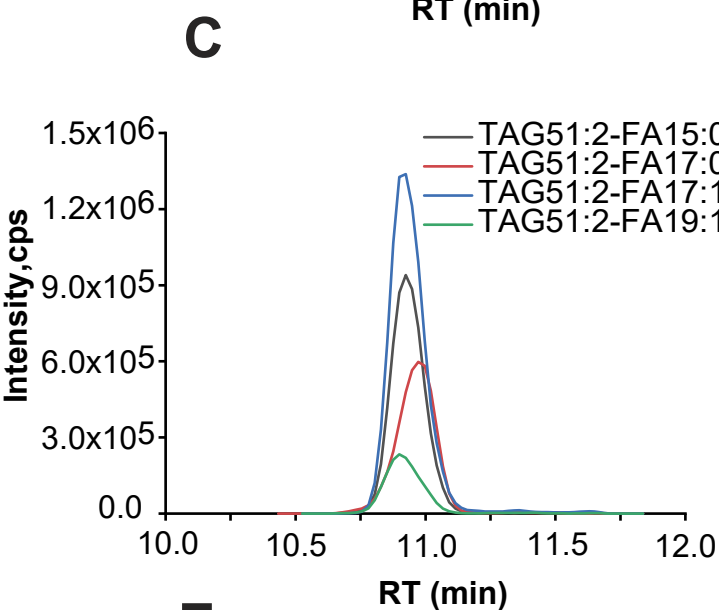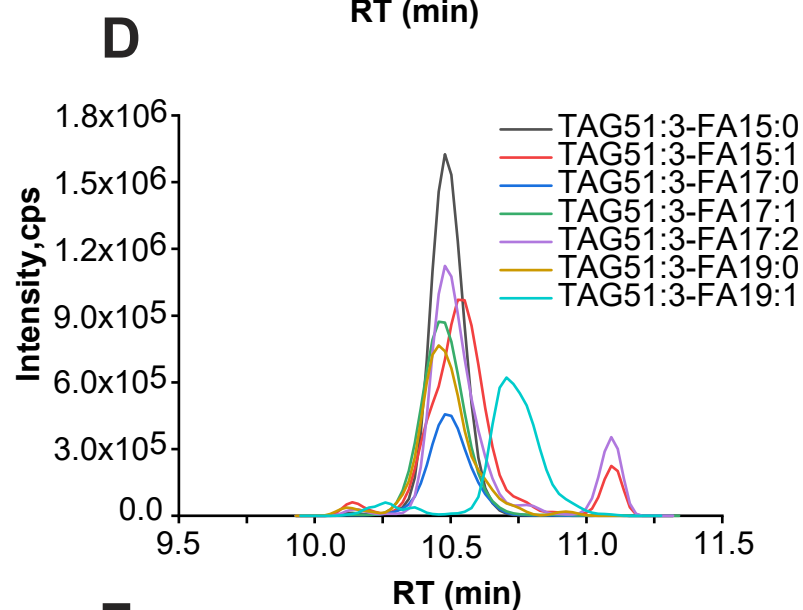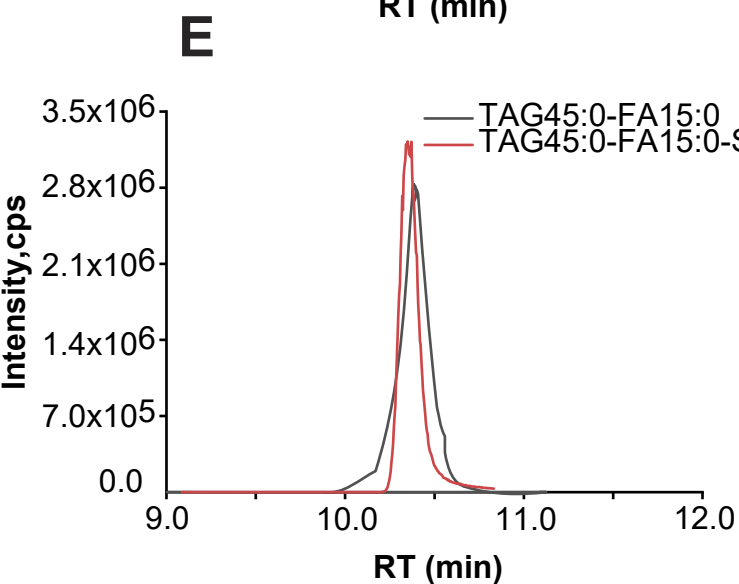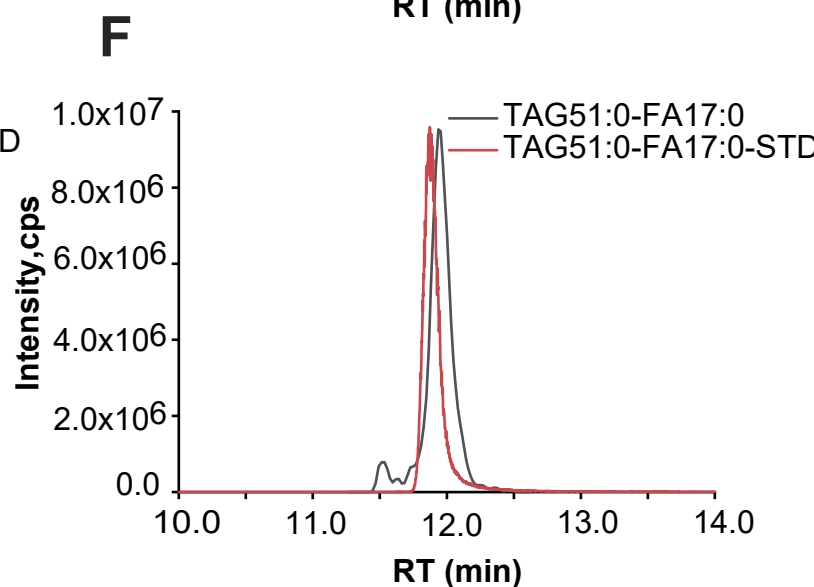

Supplement: Fig S2 [file mmc2.pdf]

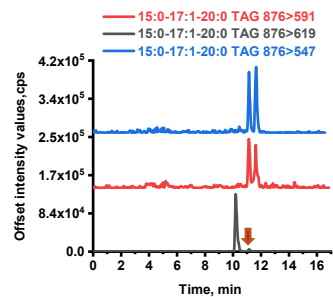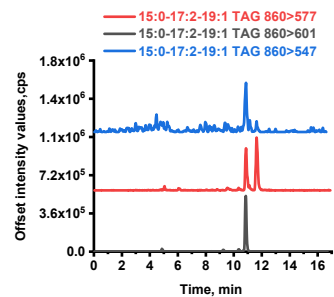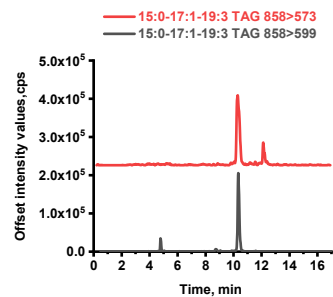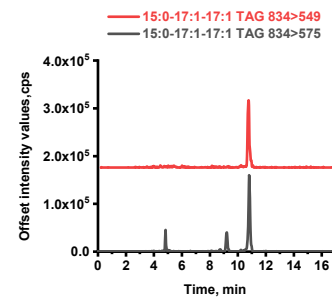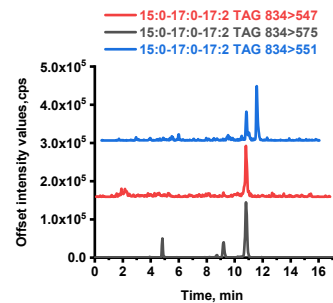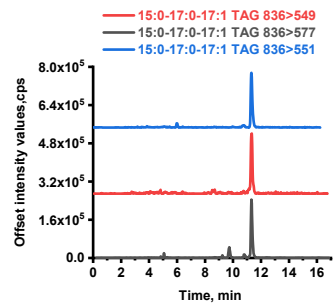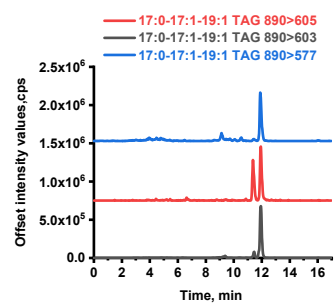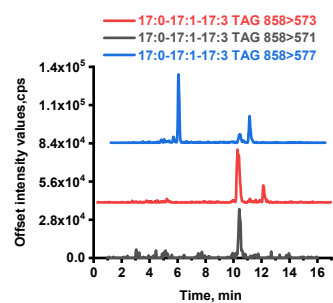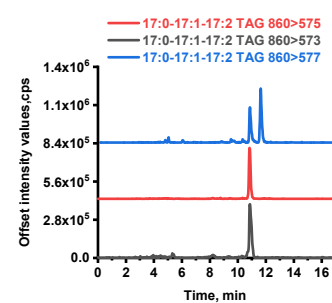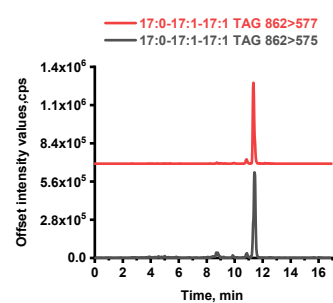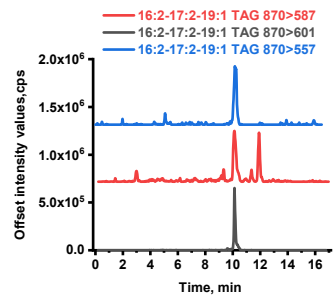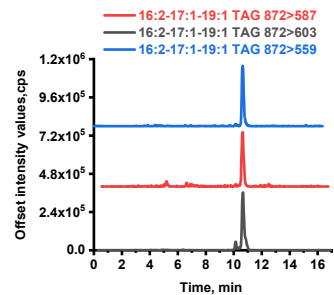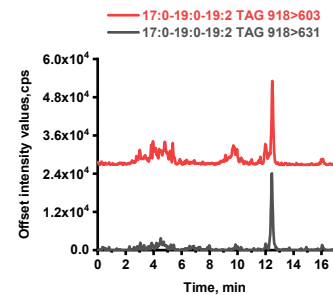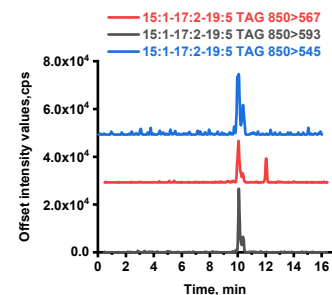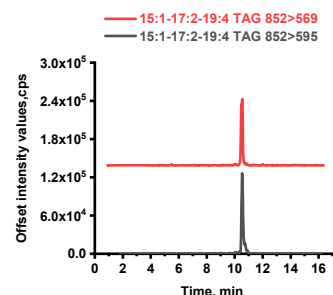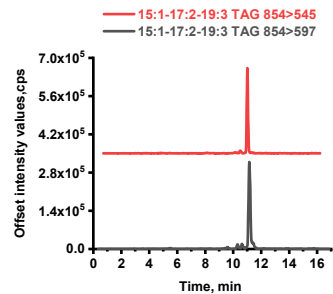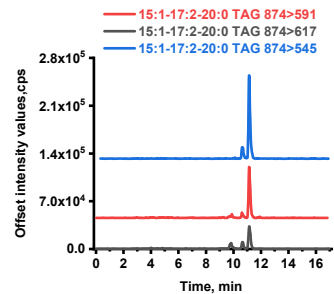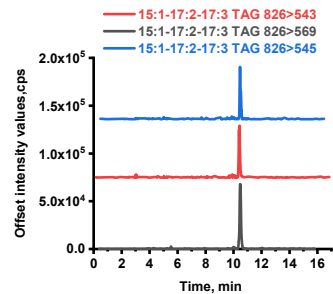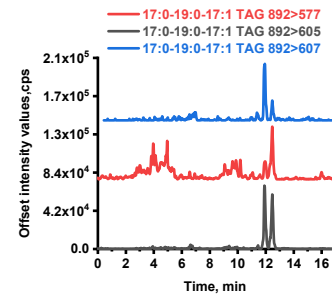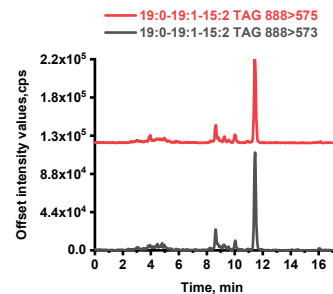

Supplement: Fig S3 [file mmc3.pdf]

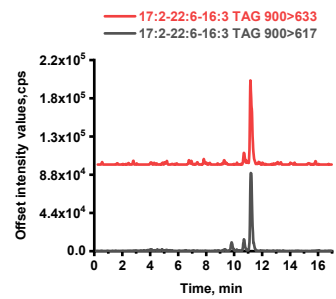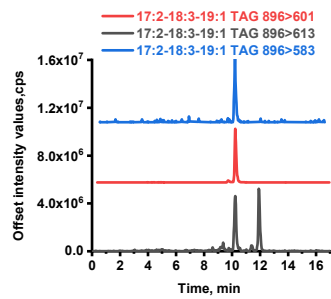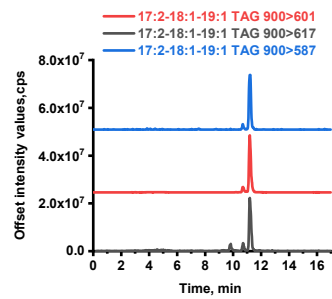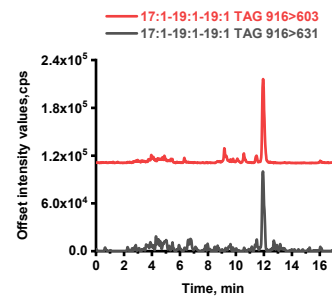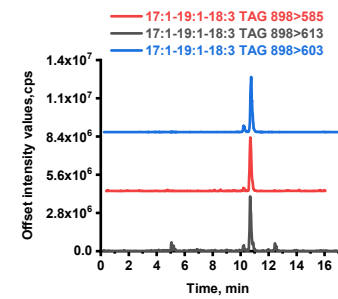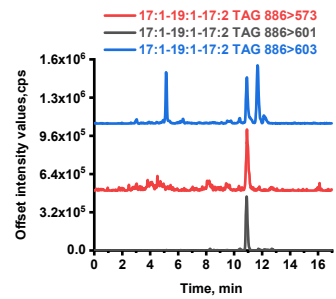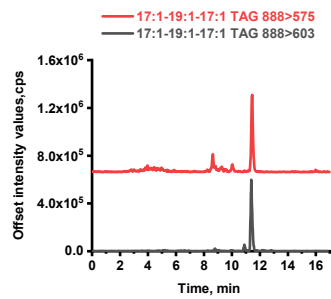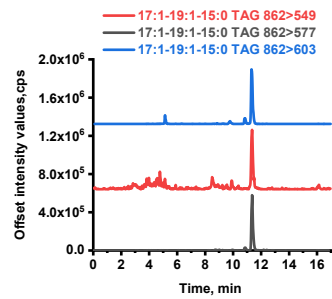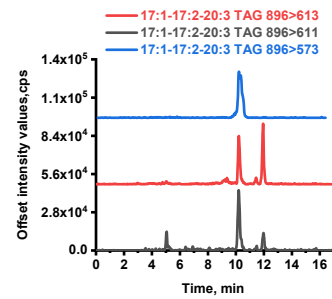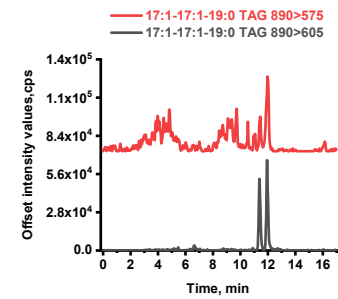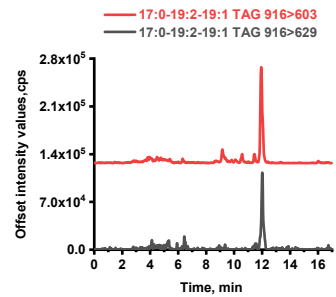

Supplement: Fig S4 [file mmc4.pdf]

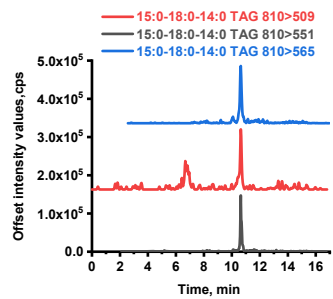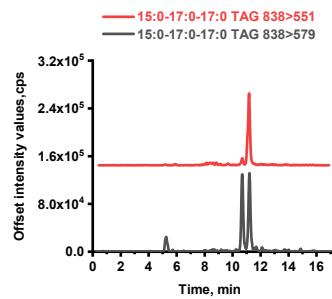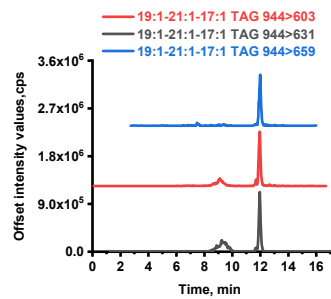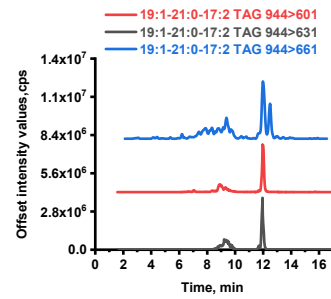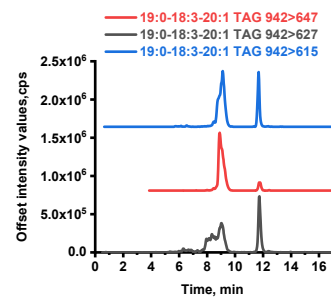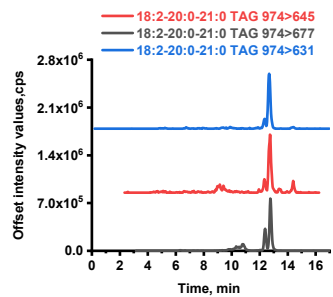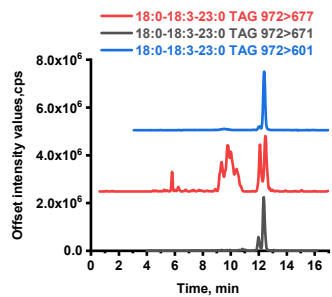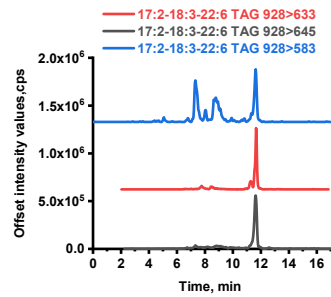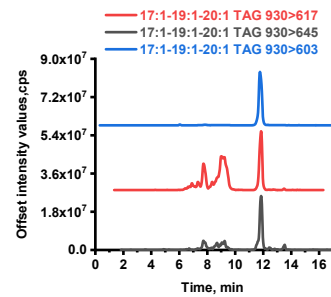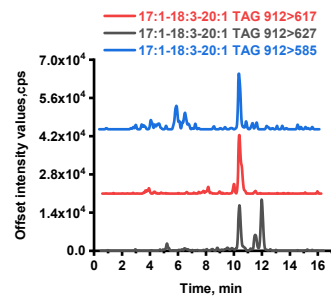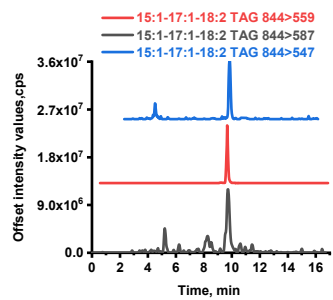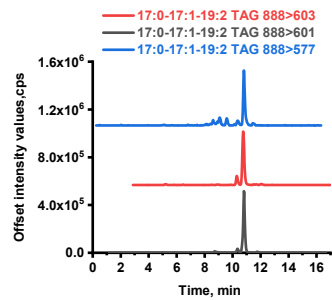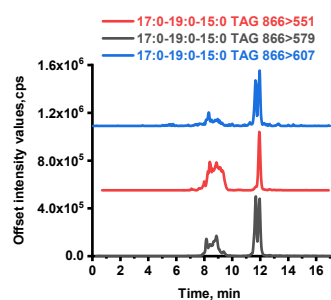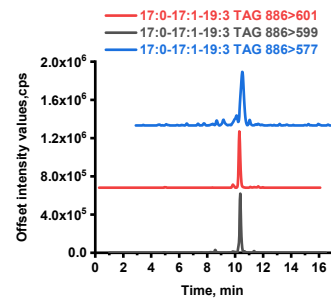

Supplement: Fig S5 [file mmc5.pdf]

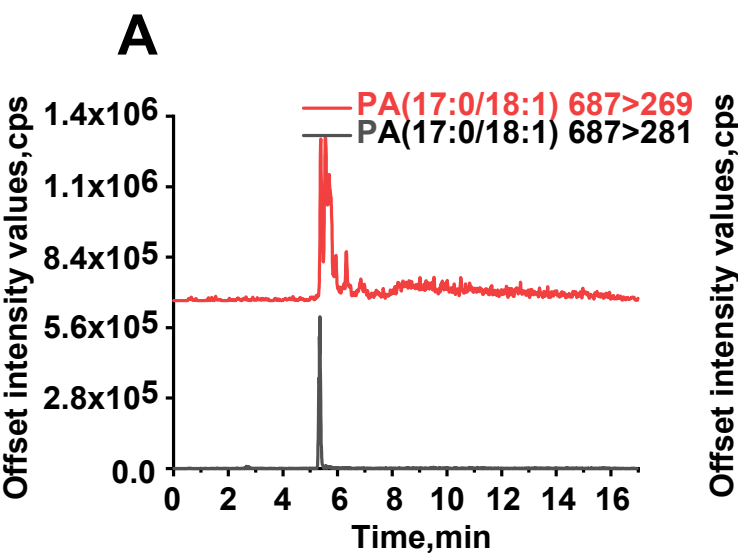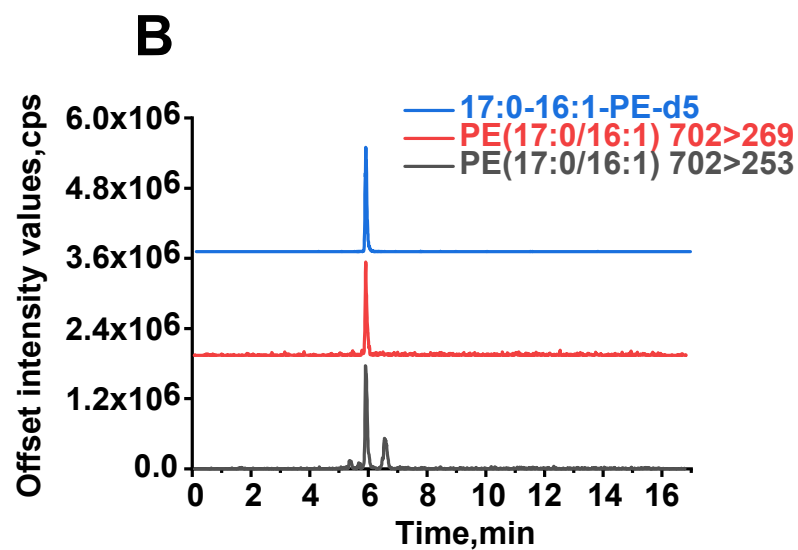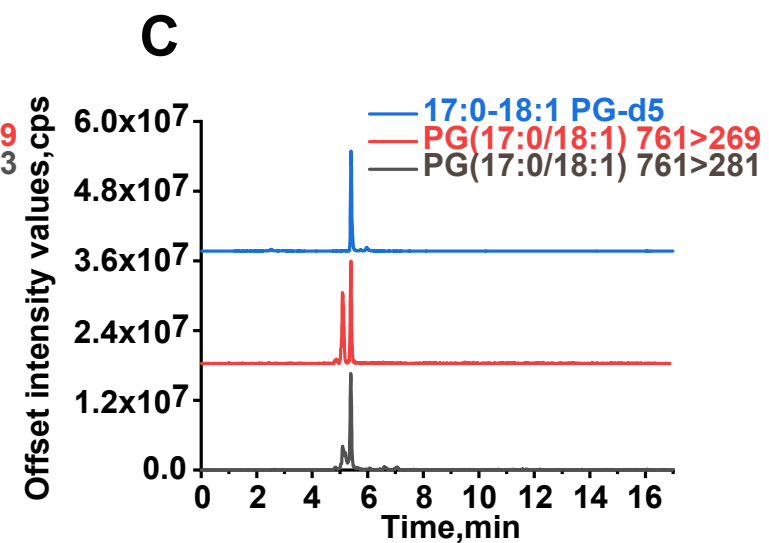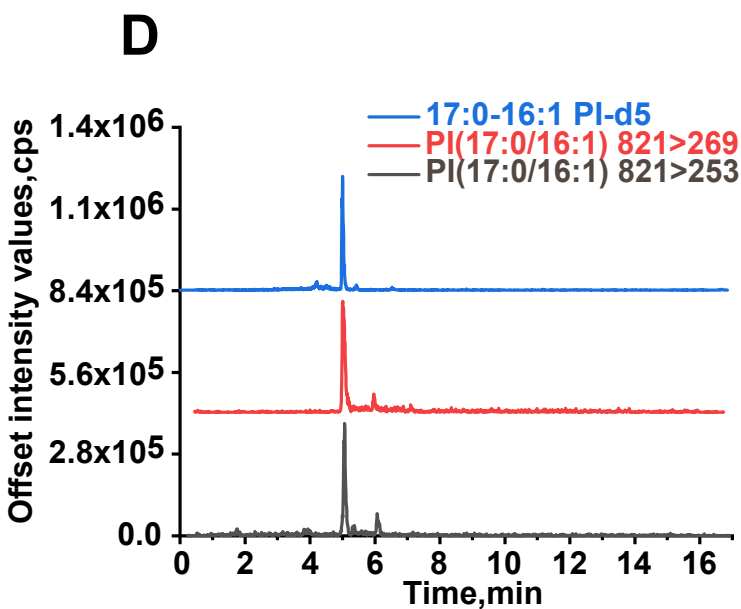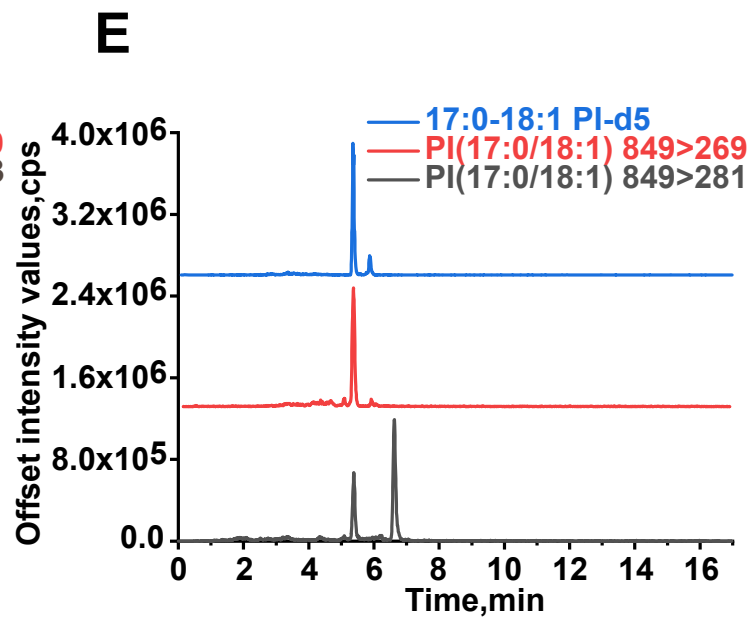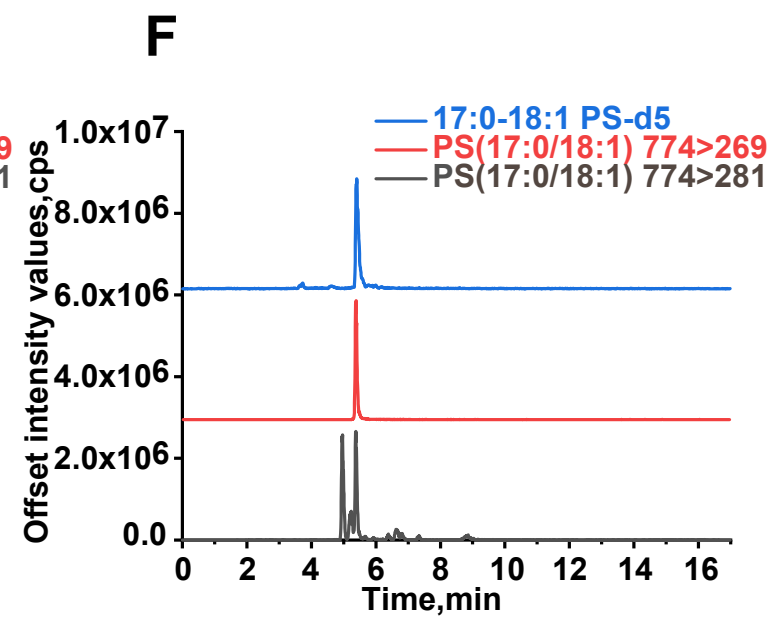

Supplement: Fig S6 [file mmc6.pdf]

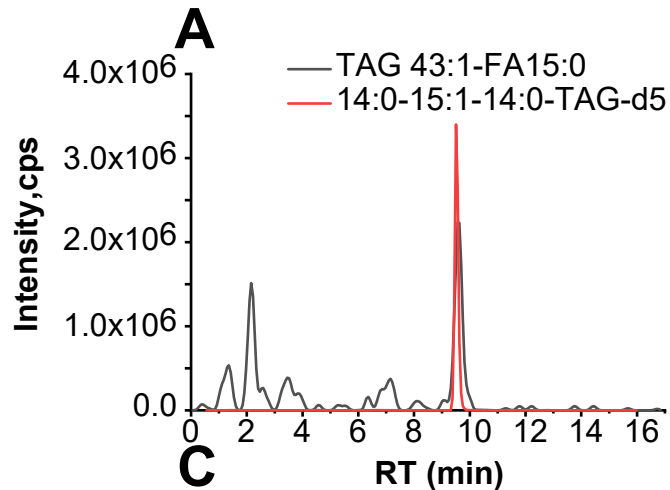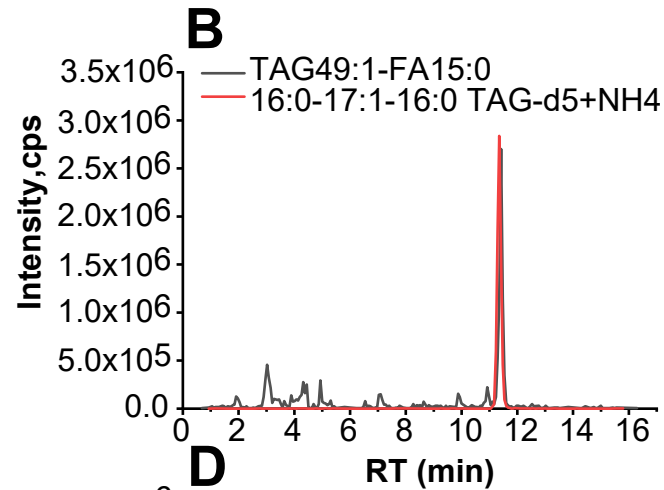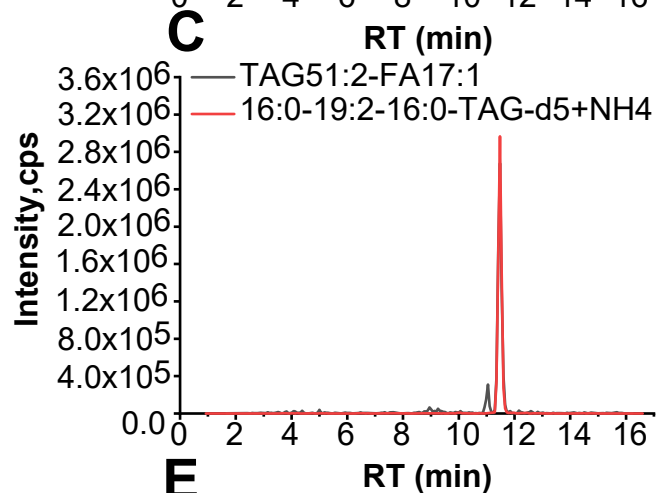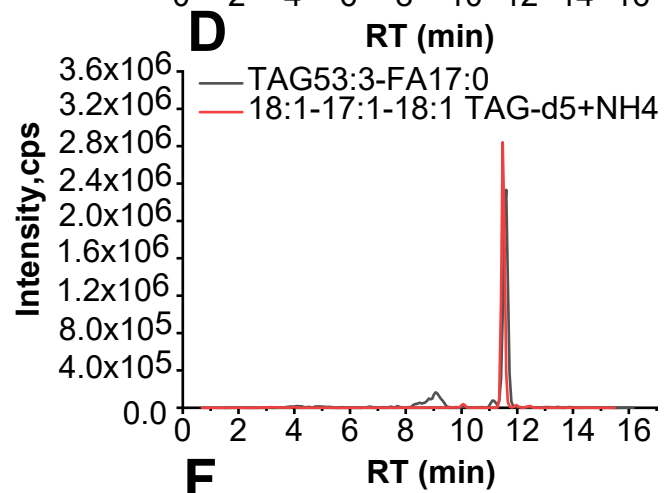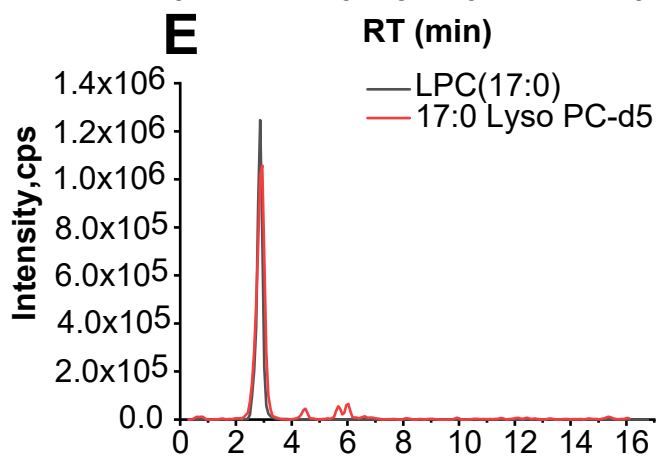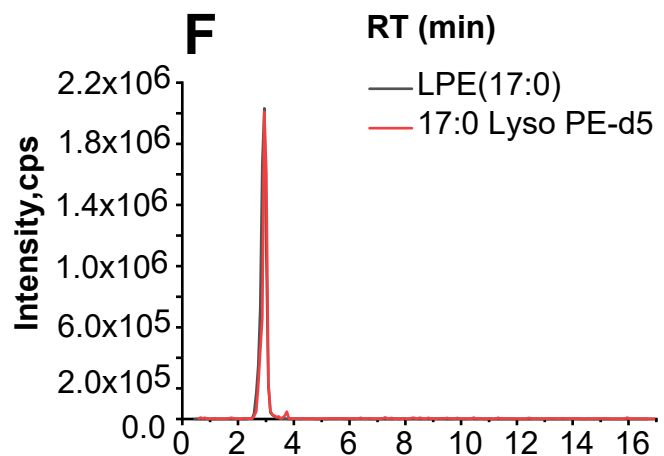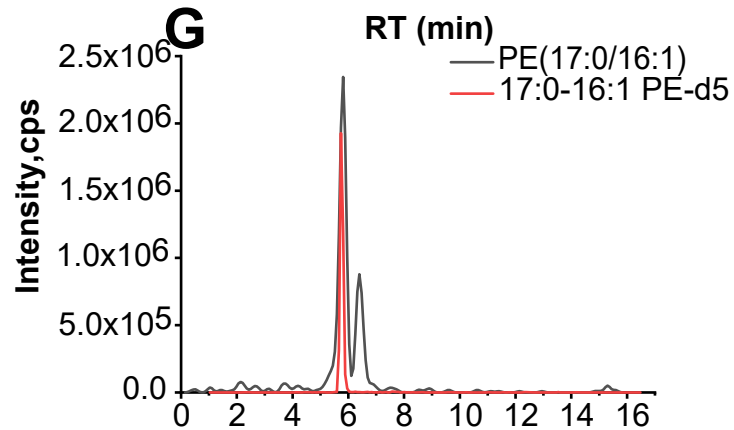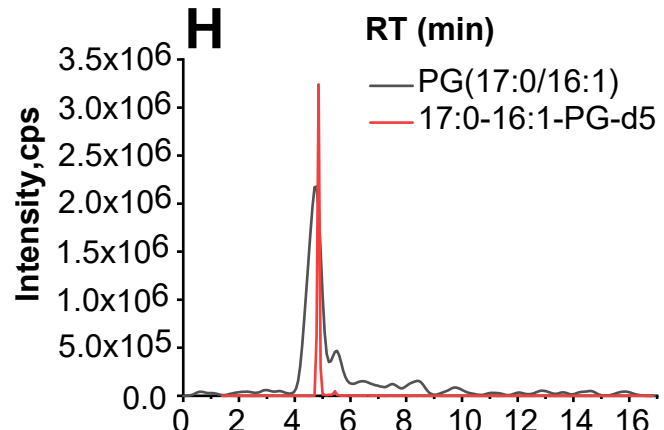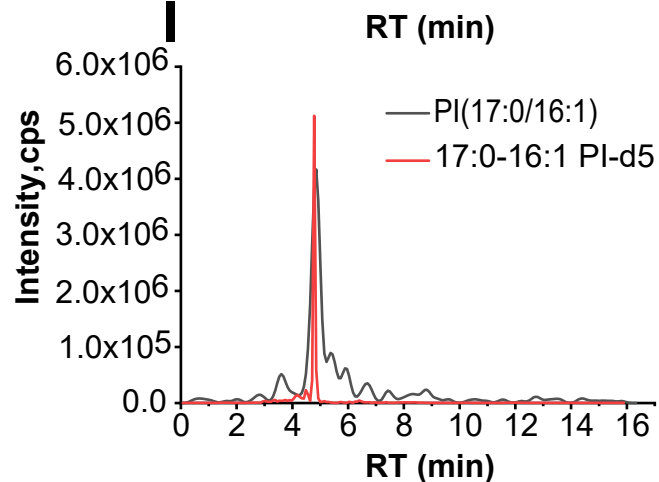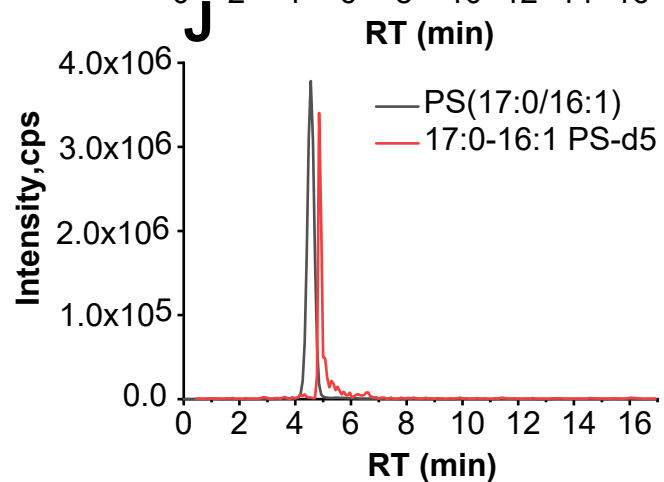

Supplement: Fig S7 [file mmc7.pdf]

**A**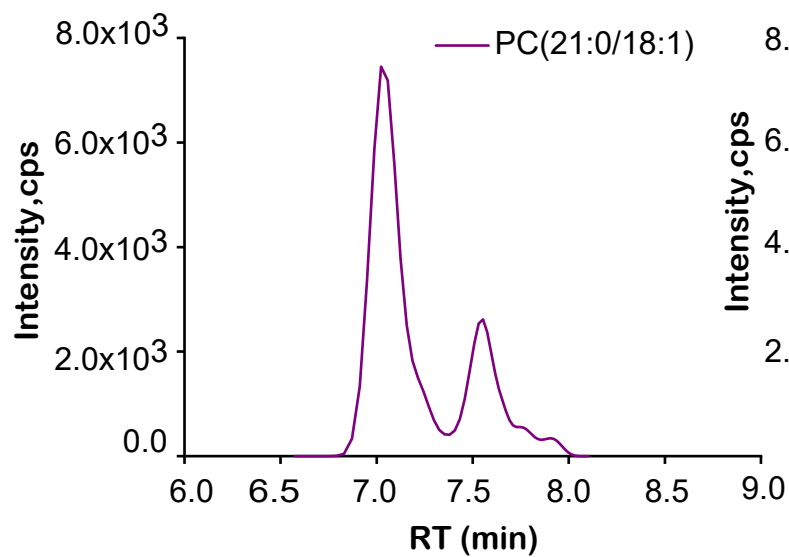**B**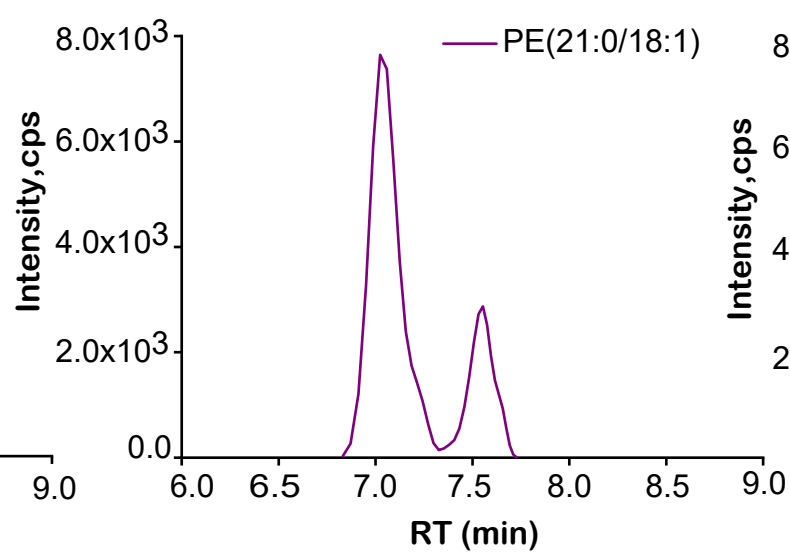**C**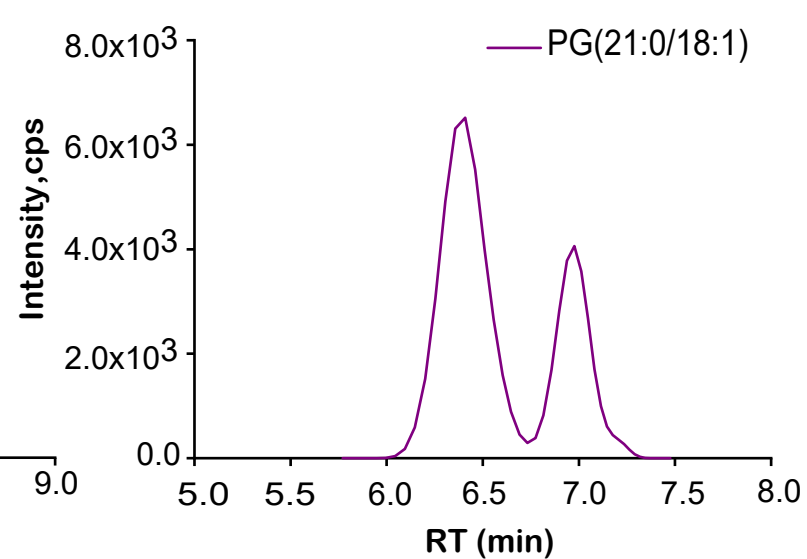**D**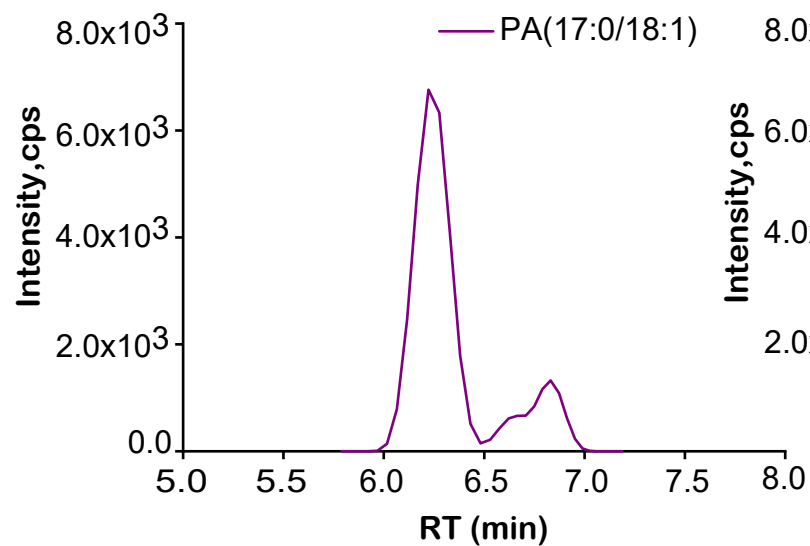**E**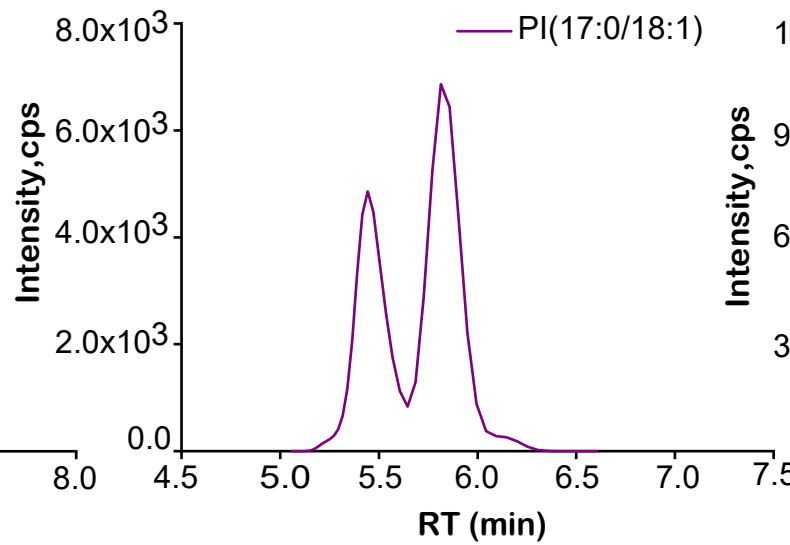**F**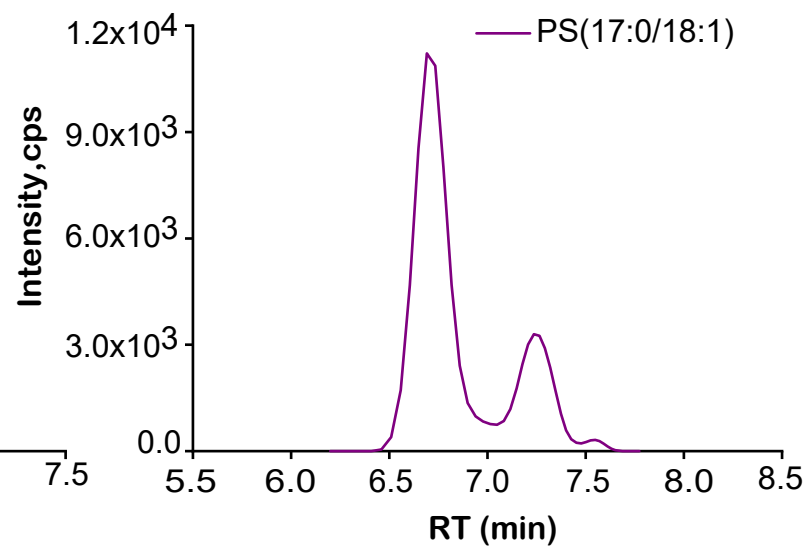

Supplement: Fig S8 [file mmc8.pdf]

**A**

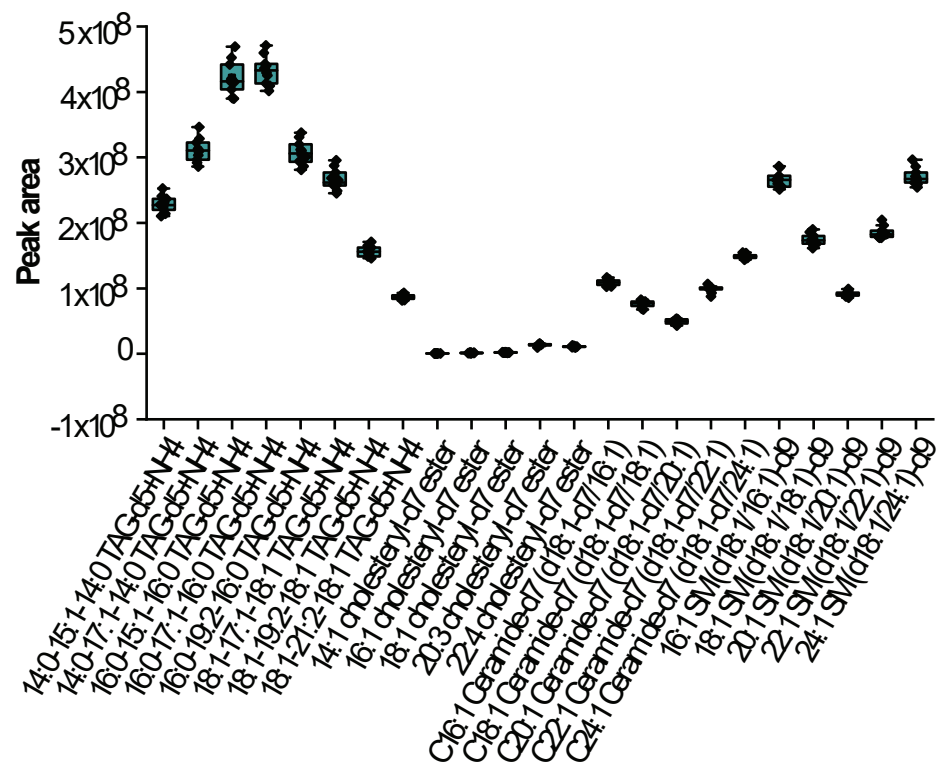

# B

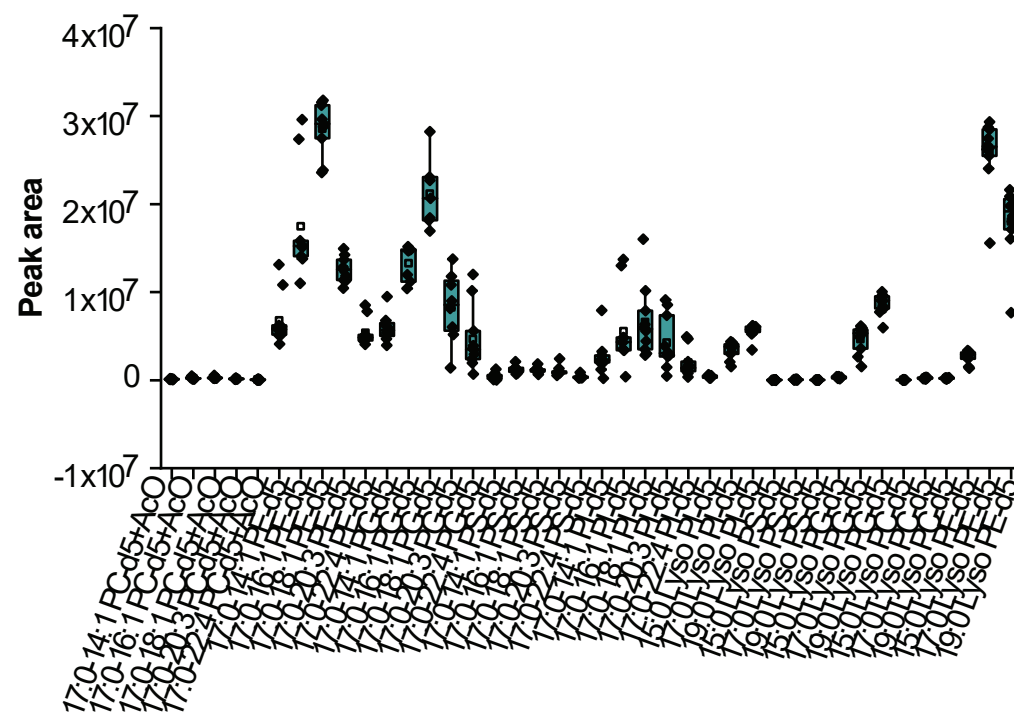

Supplement: Fig S9 [file mmc9.pdf]

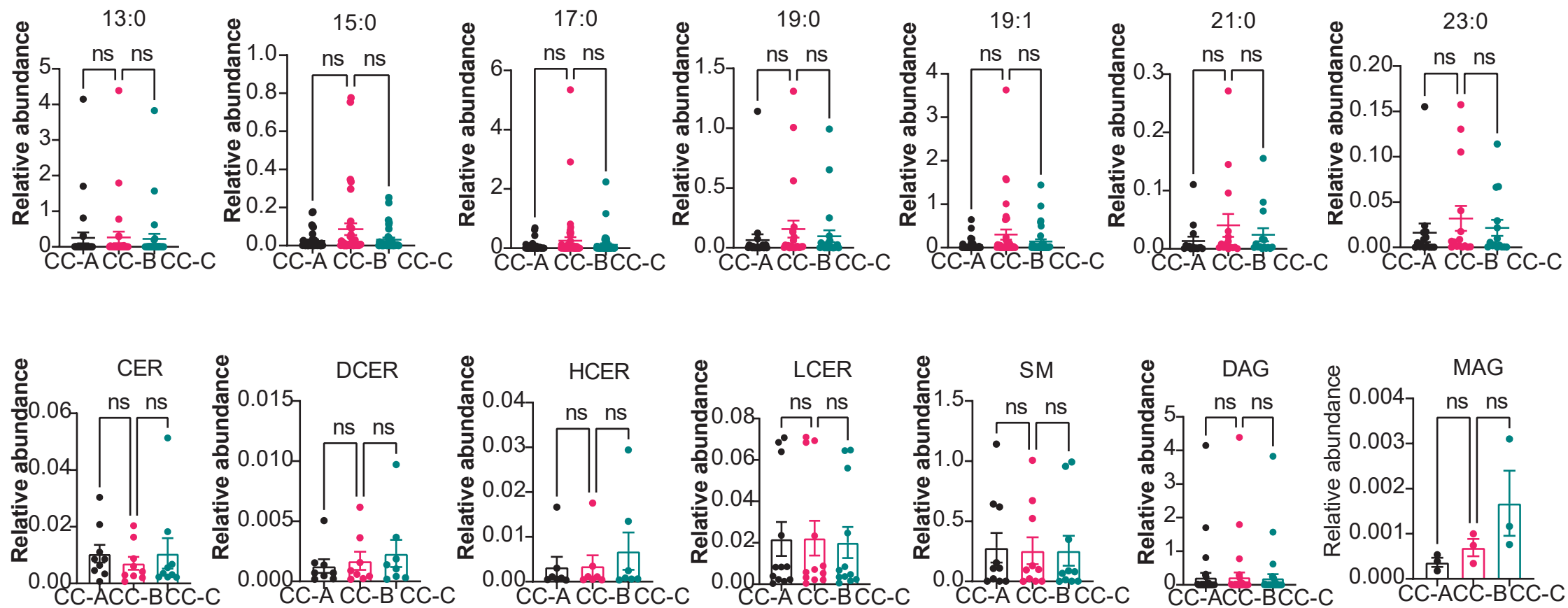

Supplement: Fig S10 [file mmc10.pdf]

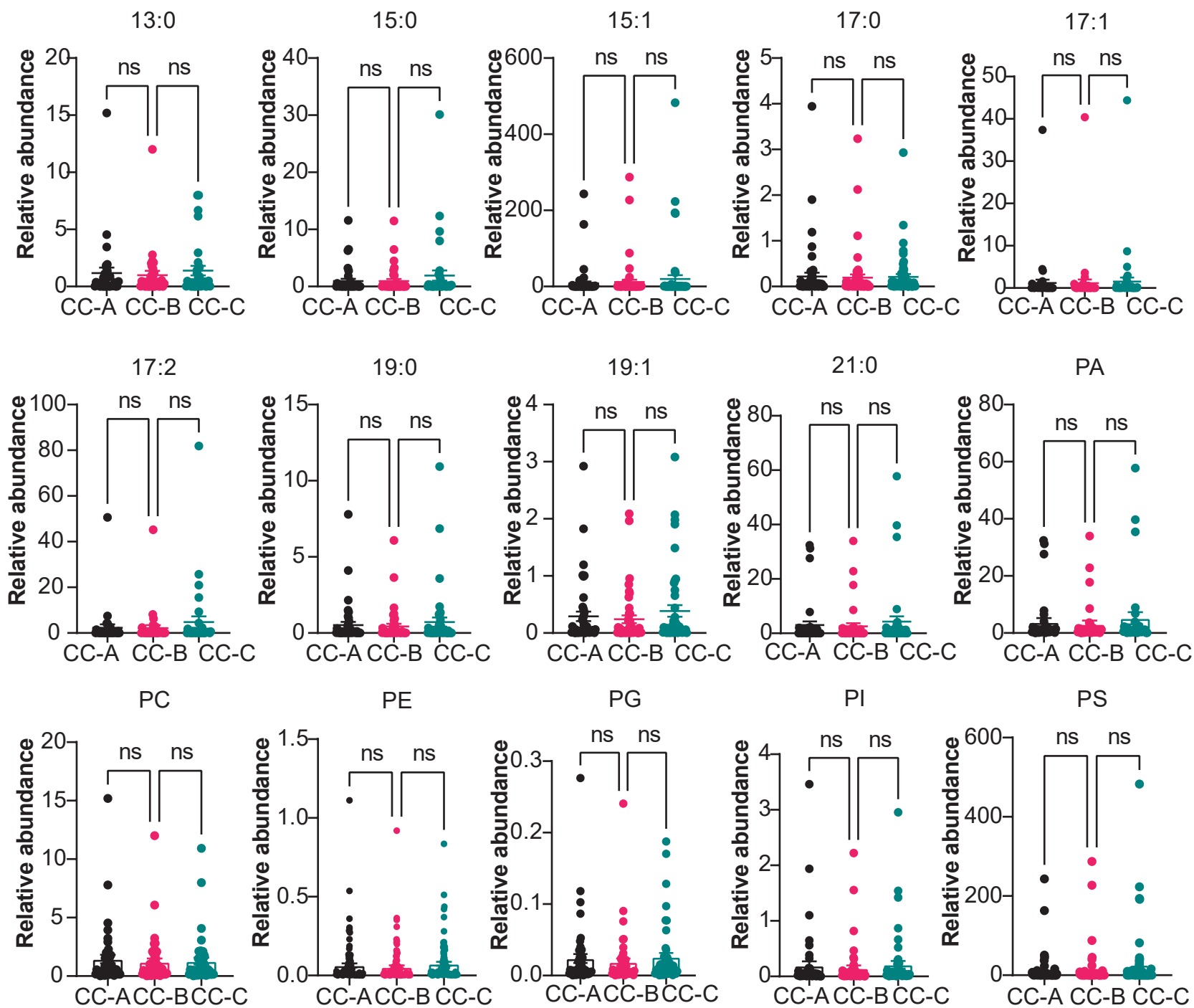

Supplement: Fig S11 [file mmc11.pdf]

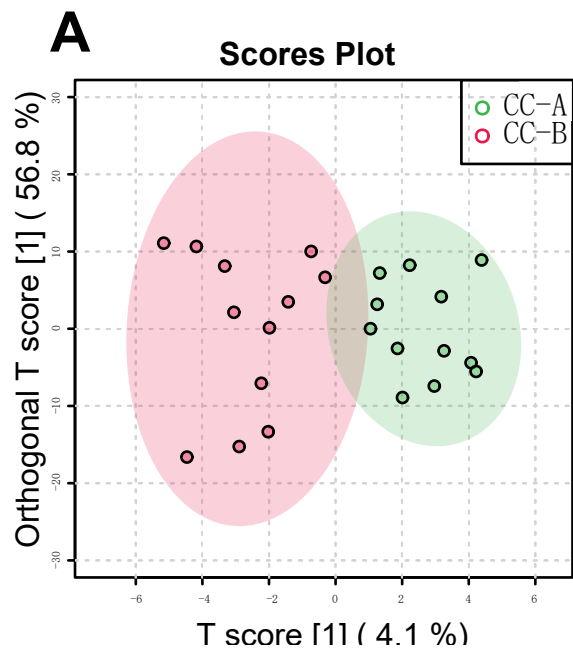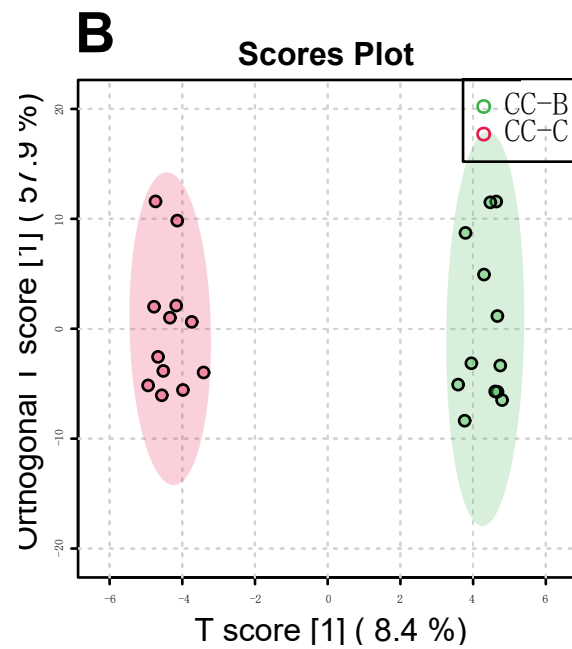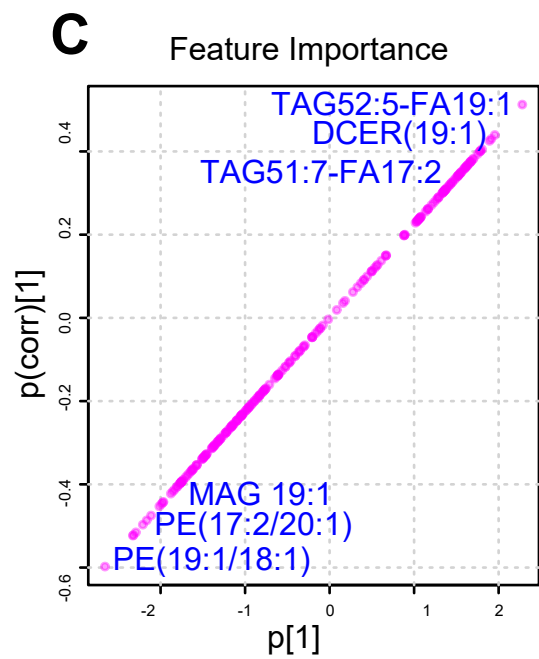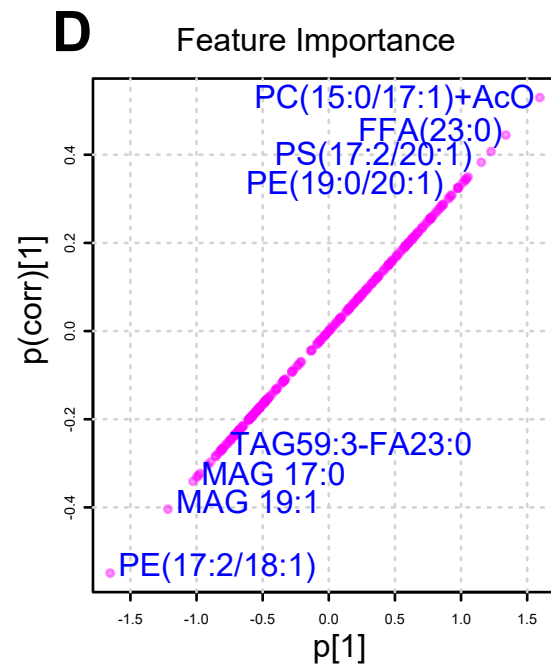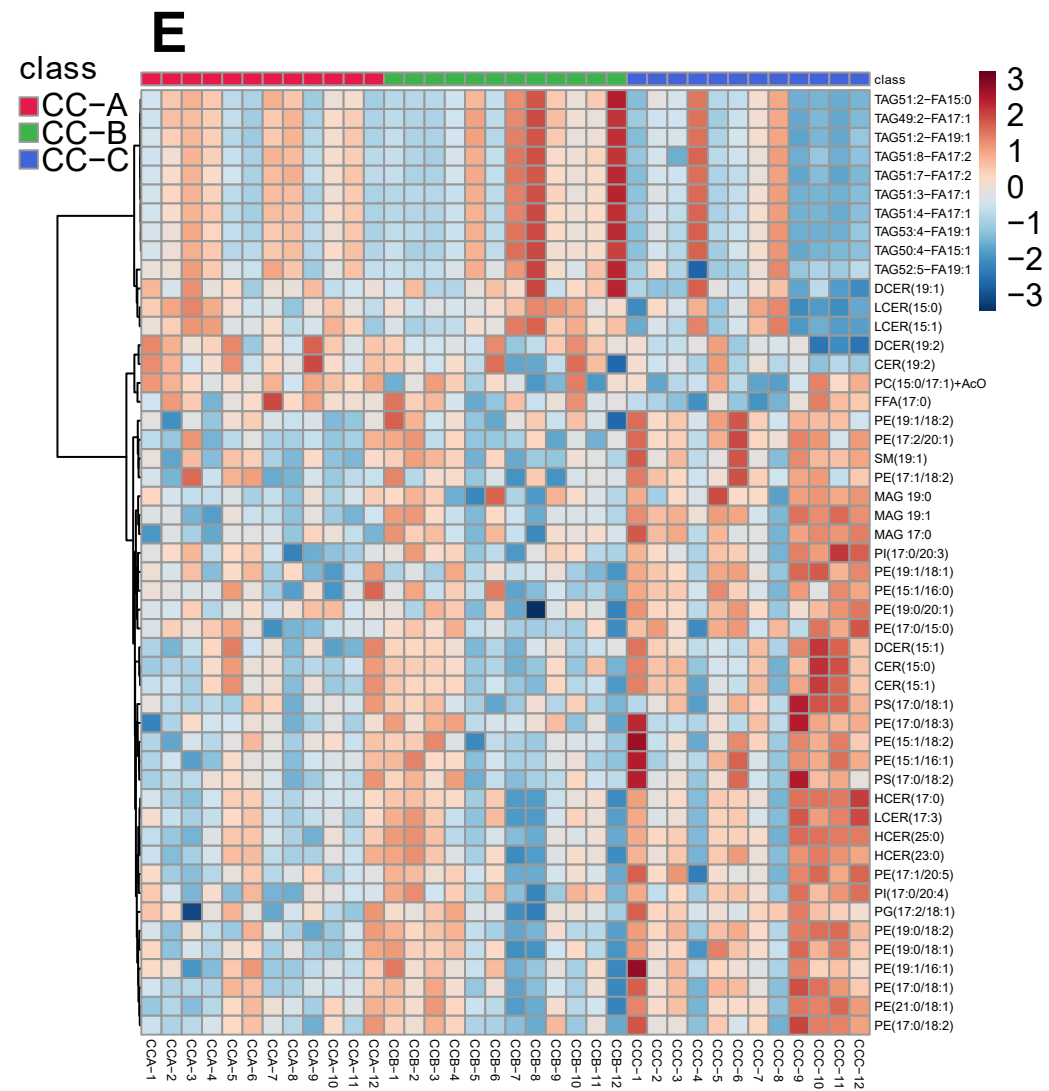

Supplement: Fig S12 [file mmc12.pdf]

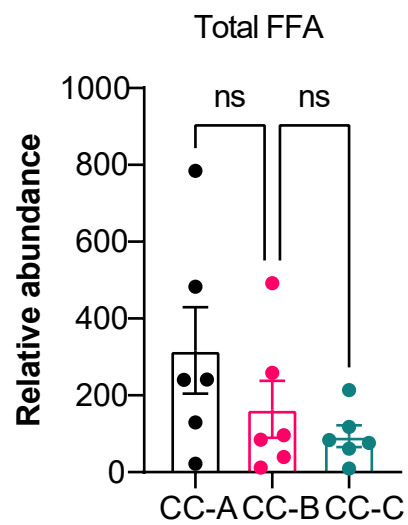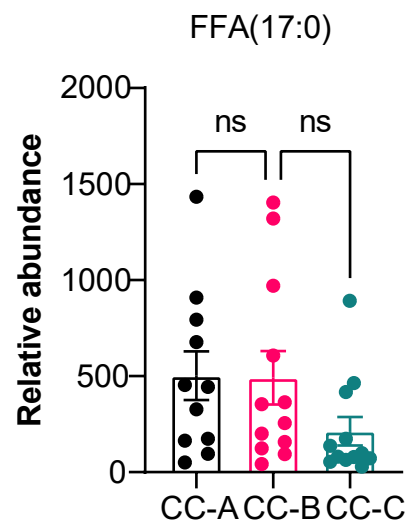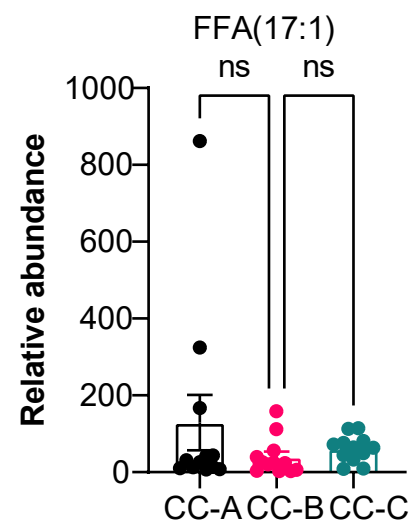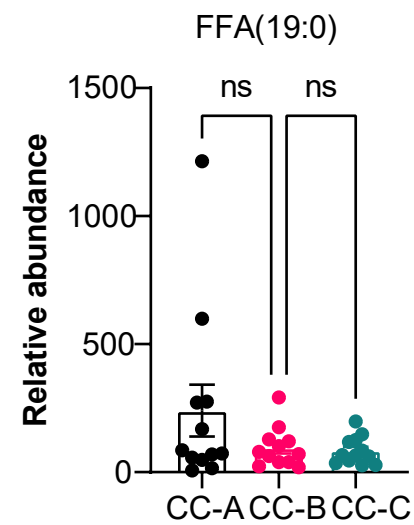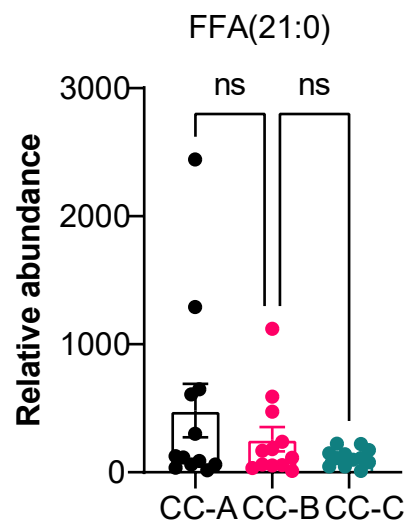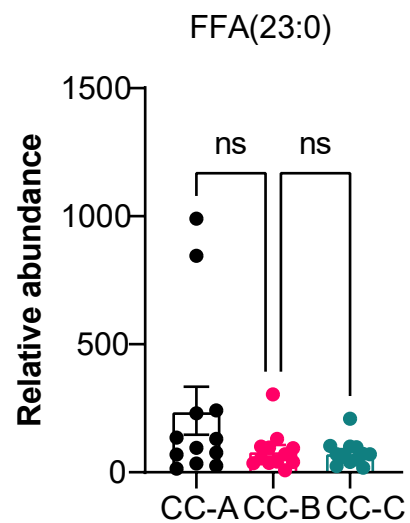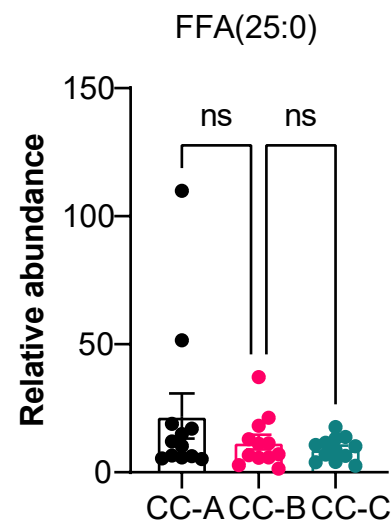

Supplement: Fig S13 [file mmc13.pdf]
